# Identification of alpha1-oleate as a potent regulator of adipokine-dependent metabolism, in bladder cancer tissue

**DOI:** 10.1186/s40170-026-00445-2

**Published:** 2026-06-23

**Authors:** Farhan Haq, Siddharth Chinchankar, Ines Ambite, Shahram Ahmadi, Hien Tran, Atefeh Nazari, Antonin Brisuda, Jaromir Hacek, Marek Babjuk, Catharina Svanborg

**Affiliations:** 1 Department of Microbiology, Immunology and Glycobiology (MIG), Institute of Laboratory Medicine, Lund University, Klinikgatan 28, BMC B13, Lund 222 42, Lund, Sweden; 2 Department of Biosciences, COMSATS University, Islamabad, Pakistan; 3 Department of Urology, Motol University Hospital, 2nd Faculty of Medicine, Charles University Praha, Prague, Czech Republic; 4 Department of Pathology, Motol University Hospital, 2nd Faculty of Medicine, Charles University Praha, Prague, Czech Republic

**Keywords:** Alpha1-oleate, Clinical trial, Non‐muscle invasive bladder cancer, Adiponectin, Leptin, Glucose metabolism, Lipid metabolism

## Abstract

**Background:**

Metabolic dysfunctions are associated with increased cancer morbidity and rapid tumor progression. The alpha1-oleate complex has been used successfully to treat bladder cancer in a placebo-controlled Phase II study. Studies of the related BAMLET complexes recently identified effects on metabolism, suggesting additional functions of the “HAMLET family” of tumoricidal complexes.

**Aims:**

To investigate if intravesical alpha1-oleate treatment affects metabolism in bladder cancer tissue.

**Materials and methods:**

Samples were obtained from patients with NMIBC, enrolled in a placebo-controlled study of intravesical alpha1-oleate treatment [1]. Cells shed into the urine were harvested at each instillation and biopsies obtained at TURBT after six instillations of alpha1-oleate. The shed cells and biopsies were subjected to RNA sequencing and genome-wide transcriptomic analysis, and urine samples were analyzed using adipokine arrays.

**Results:**

RNA sequencing detected significant inhibition of metabolic genes and networks in shed cells and tissue biopsies from alpha1-oleate treated patients, compared to the placebo group. Genes and gene networks regulating glucose and lipid synthesis were inhibited in alpha1-oleate treated patients, including *ADIPOQ*, which encodes the lipid- and glucose-regulating protein adiponectin and *LEP*, which encodes the metabolism and fat storage regulator Leptin. Leptin was further identified as an upstream regulator of the adiponectin response to alpha1-oleate and *LEP* and *ADIPOQ* networks showed strong interconnection in treated tissues. Analysis of shed tumor cells, suggested rapid kinetics of the metabolic response and urine levels of adiponectin and leptin were increased post-treatment, compared to pre-treatment samples and the shed tumor cells in urine contained adiponectin and leptin, as shown by immunohistochemistry.

**Conclusions:**

The findings identify potent local effects of alpha1-oleate treatment on tumor metabolism, after intravesical instillation. The metabolic regulators adiponectin and leptin were affected in tumor tissue and cells containing adiponectin and leptin were shed into the urine, potentially depleting the tumor of cells with high adipokine levels. These potent effects suggest a new molecular approach for targeting and inhibiting key regulators of cancer metabolism in superficial tumors.

**Trial registration:**

Doubleblinded Phase I/II clinical trial (EudraCT 201600426914 NCT03560479, Registration Date 20180521).

**Supplementary Information:**

The online version contains supplementary material available at 10.1186/s40170-026-00445-2.

## Background

Metabolic dysfunctions constitute a major health threat, associated with diabetes, obesity and cancer [2–4]. Altered lipid metabolism and obesity drive the development of cancer, by promoting cancer cell proliferation, growth, and long-term survival of cancer tissue [2]. New mechanistic insights now offer opportunities to specifically target the metabolic aspect of cancer development therapeutically, for example by interfering with specific adipokines [5–7]. Adipose tissue dysfunctions alter the secretion of adipokines such as adiponectin and leptin that regulate glucose and lipid metabolism and influence oncogenic transformation, cancer progression and risk of metastasis across multiple tumor types [8, 9]. Elevated levels of the adiponectin and leptin receptors in bladder cancer tissue compared to benign urothelium, further implicate the adiponectin–leptin axis in bladder cancer biology [10]. Adipose tissue is not commonly observed in non-muscle invasive bladder cancer, suggesting a non-adipocyte origin of these molecules [11, 12].

The tumoricidal complex HAMLET (human alpha-lactalbumin made lethal to tumor cells) has documented therapeutic efficacy in several cancer models, including bladder cancer [13–19]. The synthetic peptide-based alpha1-oleate complex, comprising the N-terminal domain of alpha-lactalbumin and oleic acid, has been used successfully to treat patients with non-muscle invasive bladder cancer (NMIBC) [1, 20, 21]. Inhibition of cancer-related gene expression accompanied the reduction in tumor number and tumor size in alpha1-oleate treated patients [20]. In a parallel study, beneficial effects of BAMLET (Bovine alpha-lactalbumin made lethal to tumor cells) were observed in models of diet-induced obesity and diabetes [22].

This study investigated the effect of intravesical alpha1-oleate treatment on tumor metabolism in patients with bladder cancer, using samples obtained in a prospective Phase II study [1, 20]. Alpha1-oleate treatment caused pronounced metabolic changes in treated tissues compared to placebo. Adiponectin and Leptin were identified as strongly affected and as regulators of metabolic functions in treated tumors. In contrast, urine samples, collected to investigate the acute response to treatment, showed increased adipokine levels in urine and in shed tumor cells. The potent inhibition of metabolism-related genes included those relevant for bladder cancer [20], suggesting that in addition to the known tumoricidal and immune activating effects of alpha1-oleate, this complex also inhibits key metabolic functions in bladder cancer tissue.

## Materials and methods

### Clinical study

This study represents a secondary analysis from a previously published clinical trial [1, 20]. The single-center, placebo-controlled, double-blinded Phase I/II clinical trial (EudraCT 2016-004269-14; NCT03560479) evaluated intravesical alpha1-oleate in patients with NMIBC scheduled for TURBT. The study was approved by the State Institute for Drug Control (SUKL) in the Czech Republic; number 273,799/17-I and the Ethics Committee of the Motol University Hospital; number EK-786/17, with all participants providing informed consent. Demographic, clinical, and tumor characteristics were documented in electronic case report forms (eCRF) and were monitored by an external monitor. Patients received six intravesical instillations of alpha1-oleate (8.5 mM; *n* = 14 tumors) or placebo (*n* = 27 tumors) over one month before surgery, and all completed treatment.

The inclusion criteria were patients with non-muscle invasive papillary bladder cancer (NMIBC) identified by cystoscopy and awaiting TURB, with women of childbearing potential having a negative pregnancy test and using appropriate contraception, and all patients able to retain bladder contents for at least 1 h. The exclusion criteria was patients with history of muscle invasive bladder cancer, NMIBC recurrence within 6 months of prior TURB, prior intravesical BCG or chemotherapy within 12 months, other malignancies within 5 years (except of skin basaliomas), acute urinary tract infection, prior radiotherapy or systemic chemotherapy, use of investigational agents within 1 month, concurrent illnesses affecting compliance, or previous enrollment in the trial.

Primary endpoints included safety, tumor size change, and tumor-cell shedding in urine, while secondary endpoints assessed tumor histopathology, alpha1-oleate uptake, apoptosis induction, and treatment-related gene-expression effects.

### Transcriptomics analysis

RNA was isolated from biopsy specimens and prepared using Illumina TruSeq Stranded mRNA Library Prep Kit (20020594), libraries were multiplexed and sequenced using NextSeq 500/550 High Output Kits (v2.5 2 × 75 Cycles) with an average of 22 million reads per sample. Raw sequencing data was demultiplexed using bcl2fastq (version 2.18) and RSEM (1.3) was used for abundance estimation using the human genome release 37/Ensemble 75. Samples were thoroughly QCed and visualized using dimensionality reduction (i.e. PCA), MA-plots as well as RNA-seq intrinsic biases (such as GC bias, transcriptome complexity and alignment quality). Differential expression analysis was performed using R (version 4.3.0) and the limma and DESeq2 packages. Fold changes were calculated by comparing tumors in the treated to the placebo group. Relative expression levels were analyzed and genes with an absolute fold change > 2.0 and *P* < 0.05 were considered as differentially expressed. Differentially expressed genes were functionally characterized into cancer and metabolism associated genes using the Ingenuity Pathway Analysis version 153,384,343, build 9.0 (IPA, Qiagen) software, which combines established information about overall gene functions.

RNA was further isolated from around 30 mL urine using the ZR Urine RNA Isolation Kit (Zymo Research Cat# R1039). RNA purification followed the manufacturer’s protocol with a -80 °C storage of the lysed and RNA stabilized cells. Gene expression analysis on microarray was performed by Eurofins Genomics using WT Pico amplification kit and Human Clariom S HT arrays (ThermoFisher). Data was normalized using the Robust multi-array average (RMA) algorithm in Transcriptome Analysis Console (v4.0.1.36). Differentially expressed genes comparing samples collected post-treatment to samples collected pre-treatment, were identified by ANOVA with empirical Bayes (threshold: FC ≥ 5, *P* < 0.05). Functional and pathway analyses were performed using Ingenuity Pathway Analysis version 153,384,343, build 9.0 (IPA, Qiagen) Core Analysis.

### Urine adipokine analysis

We utilized assay to screen for 16 human adipokine proteins (Adiponectin/Acrp30, C-Reactive Protein, Calbindin D, Cathepsin S, CXCL2/GRO beta/MIP-2/CINC-3, DPPIV/CD26, IGFBP-rp1/IGFBP-7, Insulin, Leptin/OB, M-CSF, Myeloperoxidase (MPO), PBEF/Visfatin, Resistin, TIMP-1, TREM-1, Vitamin D BP (encoded by *GC*)), by quantifying the concentration of each marker in urine (Cat no. LXSAHM-16). Thawed urine samples were mixed and analyzed undiluted, with all liquid handling performed using reverse pipetting, and all standards, high/low controls, and buffer controls run in duplicate. Assays followed the manufacturer’s instructions: standards and controls were reconstituted in diluent RD6-65, equilibrated, and used to generate a seven-point threefold serial dilution curve. Samples, standards, and controls (50 µL) were added to wells along with diluted Microparticle Cocktail, followed by a 2-hour incubation, magnetic washing, a 1-hour incubation with diluted Biotin–Antibody Cocktail, repeated washing, and a 30-minute incubation with diluted Streptavidin–PE. After a final wash, microparticles were resuspended in Wash Buffer and plates were read within 90 min on a Luminex or Bio-Rad analyzer, ensuring resuspension immediately before acquisition. All assays complied with manufacturer validation criteria, including sensitivity, intra-assay precision, linearity, and < 0.5% cross-reactivity or interference.

### Adiponectin and Leptin staining of shed cells in urine samples

Shed cells from urine were stained with specific antibodies against Adiponectin and Leptin. Fixed cells on cytospin slides were washed (PBS, 10 min), permeabilized (0.25% Triton X-100 in PBS, 20 min, room temperature), and then blocked (5% normal goat serum in PBS or TBS, 1 h, room temperature). Slides were then incubated with primary antibodies against Adiponectin (mouse monoclonal, 19F1, MA1-054) and Leptin (rabbit polyclonal, PA1-051) diluted 1:250 in 5% normal goat serum for 2 h at room temperature. Following primary incubation, slides were washed (0.025% Triton X-100 in PBS, 3 × 5 min) and stained with goat anti-mouse Alexa Fluor 488 (Invitrogen, A32723) and goat anti-rabbit Alexa Fluor 647 (Invitrogen, A32733) secondary antibodies (1:200, 1 h, room temperature). After secondary staining, slides were washed again (0.025% Triton X-100 in PBS, 3 × 5 min), and nuclei were counterstained with DAPI (1:1000, 15 min), followed by a final wash (3 × 1 min in PBS and 3 × 1 min in Milli-Q water). Slides were mounted using Fluoromount aqueous mounting medium (Sigma, F4680) or ProLong Glass Antifade Mountant (Invitrogen, P36980), sealed with nail polish, and imaged by laser scanning confocal microscopy (Zeiss LSM 900).

### Statistical analyses

The FDR or *P*-value for defining the differentially expressed genes in IPA in the 8.5mM alpha1-oleate -treated tumors compared to placebo was set at < 0.05. The following statistical analysis was performed using GraphPad Prism where statistical significance was defined as *P* < 0.05. The normality of the data was tested using the Shapiro-Wilk normality test, which revealed that the data was not normally distributed. The non-parametric Mann Whitney *U-*test was used to compare the median urinary adipokine levels between 8.5mM alpha1-oleate-treated and placebo-treated groups, where the statistical significance was defined as *P* < 0.05.

## Results

### Regulation of metabolic genes and functions in tumors treated with alpha1-oleate

Gene expression was analyzed in tissue biopsies from patients with bladder cancer, who were treated with the alpha1-oleate complex. Each patient received six intra-vesical instillations of alpha1-oleate for one month, and the molecular response to alpha1-oleate treatment was evaluated post treatment, by sequencing of tissue RNA obtained at transurethral resection. The gene expression profiles were compared between patients receiving alpha1-oleate or placebo.

**Fig. 1:**
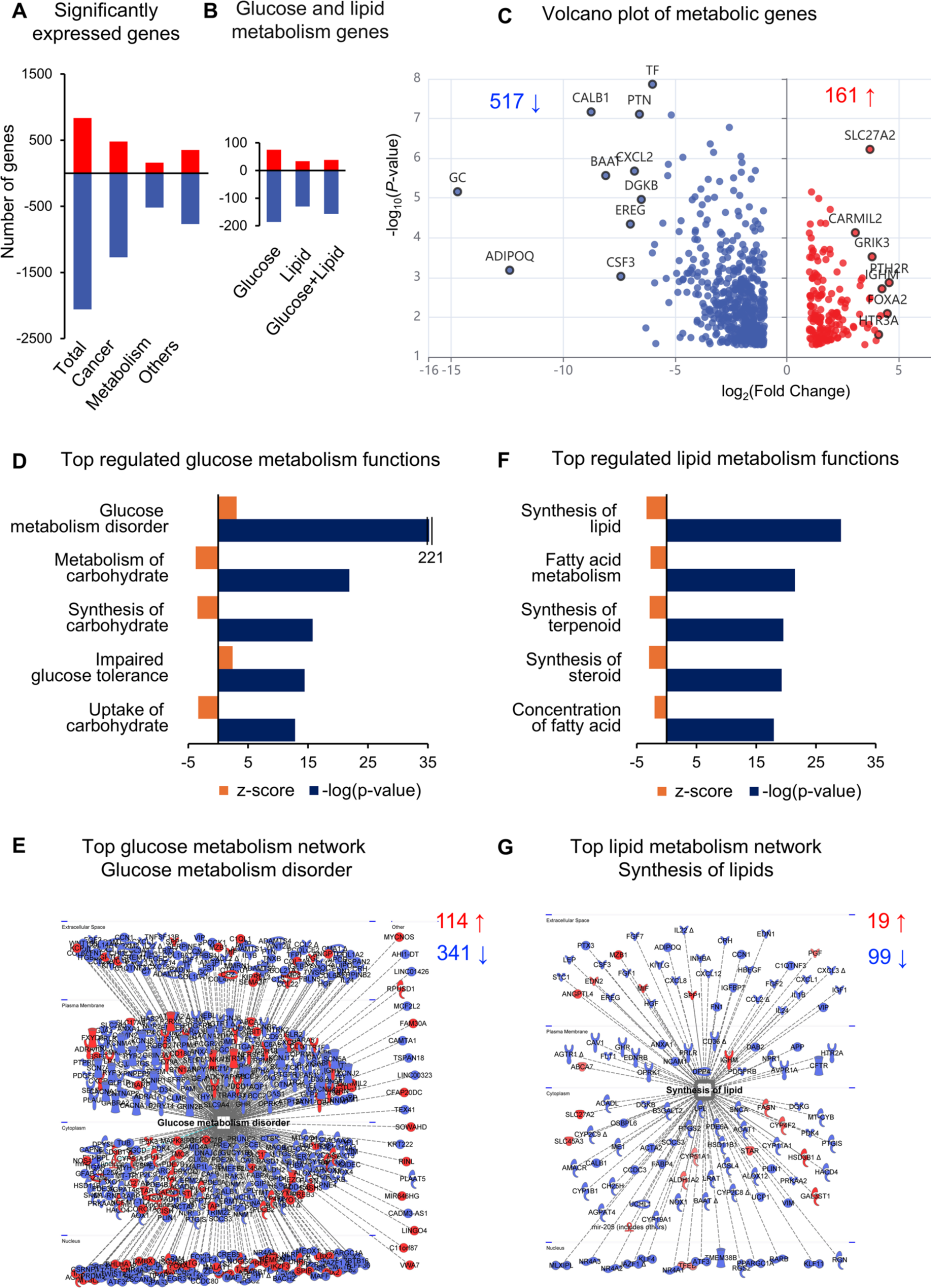
Gene expression profiling of the metabolic response to alpha1-oleate in biopsies from bladder cancer patients. (**A**) Number of differentially expressed genes in treated tumors compared to placebo (red = upregulation, blue = downregulation, FC > 2, *P* < 0.05). Major regulated functions include cancer and metabolism, with a predominance of inhibition. (**B**) Classification of significantly expressed metabolism genes in glucose metabolism, lipid metabolism or both functions. (**C**) Volcano plot of regulated metabolic genes illustrating the predominance of inhibition in alpha1-oleate treated group compared to placebo (517 downregulated and 161 upregulated genes). (**D**) Regulation of glucose metabolism functions by significantly expressed metabolism genes (orange = z-score, blue = -log(p-value)). Glucose metabolism disorder and impaired glucose tolerance are activated, and metabolism synthesis and uptake of glucose are inhibited. (**E**) Network of top regulated glucose metabolism disorder genes with inhibition of 341/445 genes (red = upregulation, blue = downregulation). (**F**) Regulation of lipid metabolism functions by significantly expressed metabolism genes. Lipid synthesis, fatty acid metabolism, synthesis of terpenoid, synthesis of steroid and concentration of fatty acid are inhibited. (**G**) Network of top regulated synthesis of lipid genes with inhibition of 99/118 genes

The inhibition of cancer-related gene expression by alpha1-oleate treatment was previously reported [1, 20]. This analysis identified effects on metabolism-associated genes, which were regulated compared to placebo (Fig. 1A, B). The significantly regulated metabolic genes (cut off foldchange (FC) > 2, *P* < 0.05), were predicted to affect glucose metabolism (261 genes), lipid metabolism (164 genes) or both functions (195 genes) (Fig. 1B). The predominance of inhibition is illustrated by the Volcano plot in Fig. 1C (517/678, 76% of significantly regulated metabolism-associated genes were inhibited in alpha1-oleate treated tumors, compared to placebo).

Functional analysis confirmed the predominance of inhibition of glucose metabolism in tumors from patients treated with alpha1-oleate compared to placebo (z-score < -2 or > 2, Fig. 1D). Glucose metabolism disorder was the most strongly affected function (341/455 downregulated genes) (Fig. 1E). In addition, glucose tolerance was predicted to be impaired, and glucose uptake, synthesis and metabolism of carbohydrates to be inhibited (Fig. 1D). Top inhibited lipid metabolism function included lipid synthesis (99/118 downregulated genes) and fatty acid metabolism, concentration of fatty acids, synthesis of terpenoid and synthesis of steroids (Fig. 1F-G).

### Alpha1-oleate inhibition of Adiponectin and genes involved in glucose and lipid metabolism

**Fig. 2:**
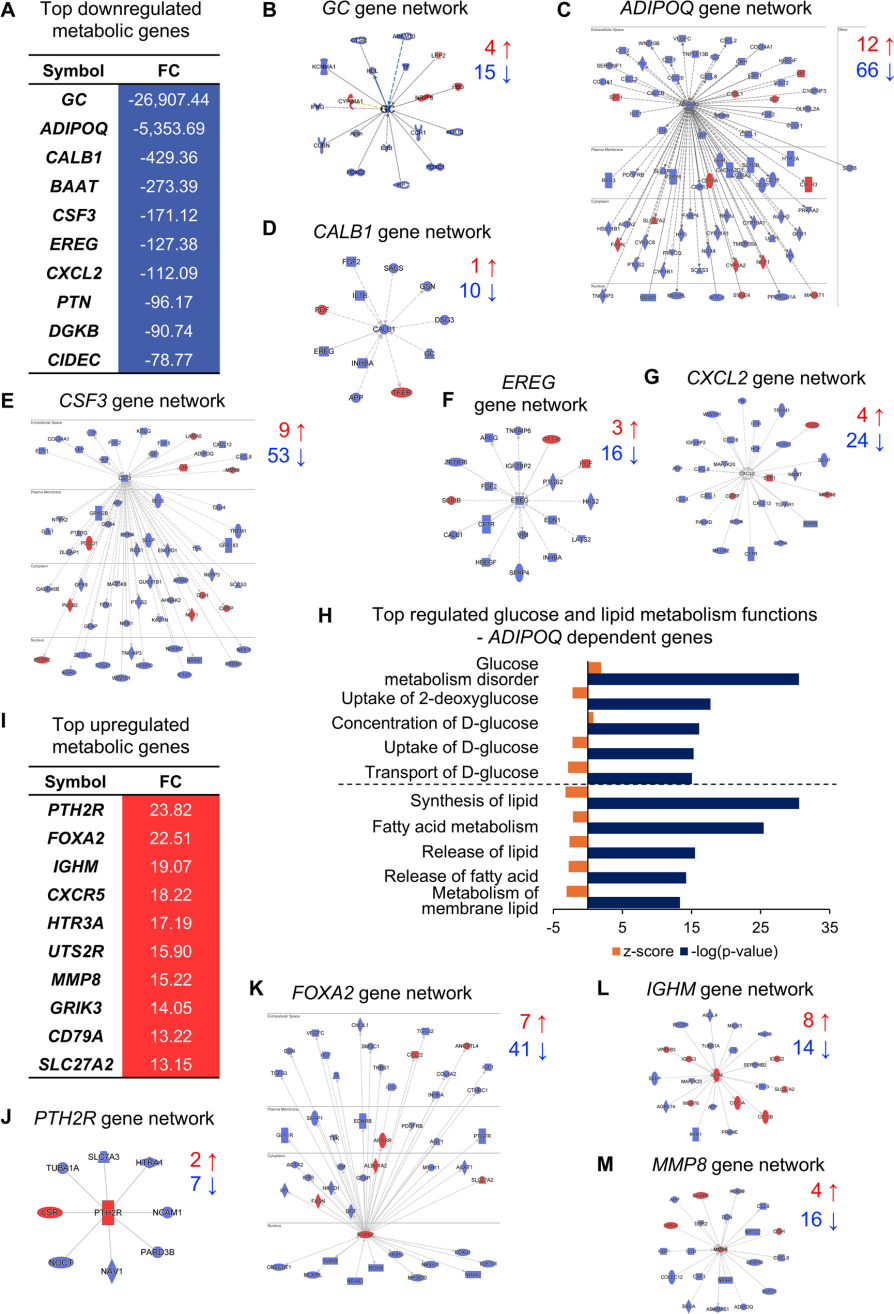
Network analysis of top regulated genes and *ADIPOQ*-dependent gene network regulation. (**A**) Top downregulated metabolic genes in treated tumors compared to placebo (FC -78 to -26900) (**B**) Network of *GC* related genes, 15/19 genes are downregulated. (**C**) Network of *ADIPOQ* related genes, 66/78 genes are downregulated. (**D**) Network of *CALB1* related genes, 10/11 genes are downregulated. (**E**) Network of *CSF3* related genes, 53/62 genes are downregulated. (**F**) Network of *EREG* related genes, 16/19 genes are downregulated. (**G**) Network of *CXCL2* related genes, 24/28 genes are downregulated. (**H**) Regulation of glucose and lipid metabolism functions by *ADIPOQ* gene network (orange = z-score, blue = -log(p-value)). A strong regulation of glucose metabolism function is observed including glucose metabolism disorder, uptake of 2-deoxyglucose, concentration of D-glucose, uptake of D-glucose and transport of D-glucose. Similarly, strong inhibition of lipid metabolism functions is observed including lipid synthesis, fatty acid metabolism, release of lipid, release of fatty acid and metabolism of membrane lipid. (**I**) Top upregulated metabolic genes in treated tumors compared to placebo (FC 13 to 24). (**J**) Network of *PTH2R* related genes, 7/9 genes are downregulated. **K**) Network of *FOXA2* related genes, 41/48 genes are downregulated. **L**) Network of *IGHM* related genes, 14/22 genes are downregulated. **M**) Network of *MMP8* related genes, 16/20 genes are downregulated

The top ten regulated metabolic genes were downregulated with FC values ranging from − 78 to -26,907, compared to placebo (Fig. 2A); including *GC* encoding the vitamin D binding GC protein (FC = -26907), *ADIPOQ* encoding Adiponectin (FC = -5354), *CALB1* encoding the calbindin 1 protein, which regulates calcium and energy metabolism (FC = -429), *BAAT* (bile acid-CoA: amino N-acyltransferase, FC = -273) and *CSF3* (colony stimulating factor 3, FC = -171). Top downregulated genes further included *EREG*, encoding the epiregulin growth factor linked to metabolic reprogramming (FC = − 127), *CXCL2*, involved in inflammation-associated metabolic signaling, (FC = − 112) and *PTN*, encoding pleiotrophin, which regulates cell growth and energy metabolism (FC = − 96), *DGKB*, encoding diacylglycerol kinase beta (FC = − 91), and *CIDEC*, a lipid droplet associated protein (FC = − 79).

The individual networks regulated by these genes are shown in Fig. 2. Inhibition predominated with 66/78 downregulated genes in the *ADIPOQ* network, 15/19 genes in the *GC* network, (Fig. 2B, C) 10/11 downregulated genes in the *CALB1* network (Fig. 2D) and 53/62 downregulated genes in the *CSF3* network (Fig. 2E). The networks of *EREG*, a metabolic growth factor, and *CXCL2*, a proinflammatory chemokine, also showed predominance of downregulation (Fig. 2F and G). The top down-regulated genes *ADIPOQ*, *EREG*,* CALB1* and *GC* formed interconnected networks (Supplementary Fig. 1), suggesting a strong functional interdependence of the top regulated genes, broadly affecting metabolism.

Functional analysis of *ADIPOQ* dependent genes predicted strong inhibition of glucose and lipid metabolism in alpha1-oleate treated tissues compared to placebo (Fig. 2H), including glucose metabolism disorder, glucose uptake, concentration and transport of glucose were strongly regulated. Lipid metabolism functions, including synthesis, release and metabolism of fatty acids and lipids were strongly downregulated in alpha1-oleate treated tissues compared to placebo (Figure H).

Paradoxically, the activated genes were shown to regulate networks of inhibited metabolic genes, including *FOXA2* (Forkhead Box A2), *PTH2R* (Parathyroid Hormone 2 Receptor), *IGHM* (Immunoglobulin Heavy Constant Mu), *CXCR5* (C-X-C Motif Chemokine Receptor 5), *HTR3A* (5-Hydroxytryptamine Receptor 3 A), *UTS2R* (Urotensin 2 Receptor), *MMP8* (Matrix Metallopeptidase 8), *GRIK3* (Glutamate Ionotropic Receptor Kainate Type Subunit 3), *CD79A* (CD79a Molecule), and *SLC27A2* (Solute Carrier Family 27 Member 2) (FC range ~ 13 to 24), suggesting that an activation of repressors might contribute to the inhibitory effect of alpha1-oleate, compared to placebo (Fig. 2I-M and Supplementary Fig. 2).

### Alpha1-oleate regulation of metabolism via a *LEP*/*ADIPOQ* nexus

**Fig. 3:**
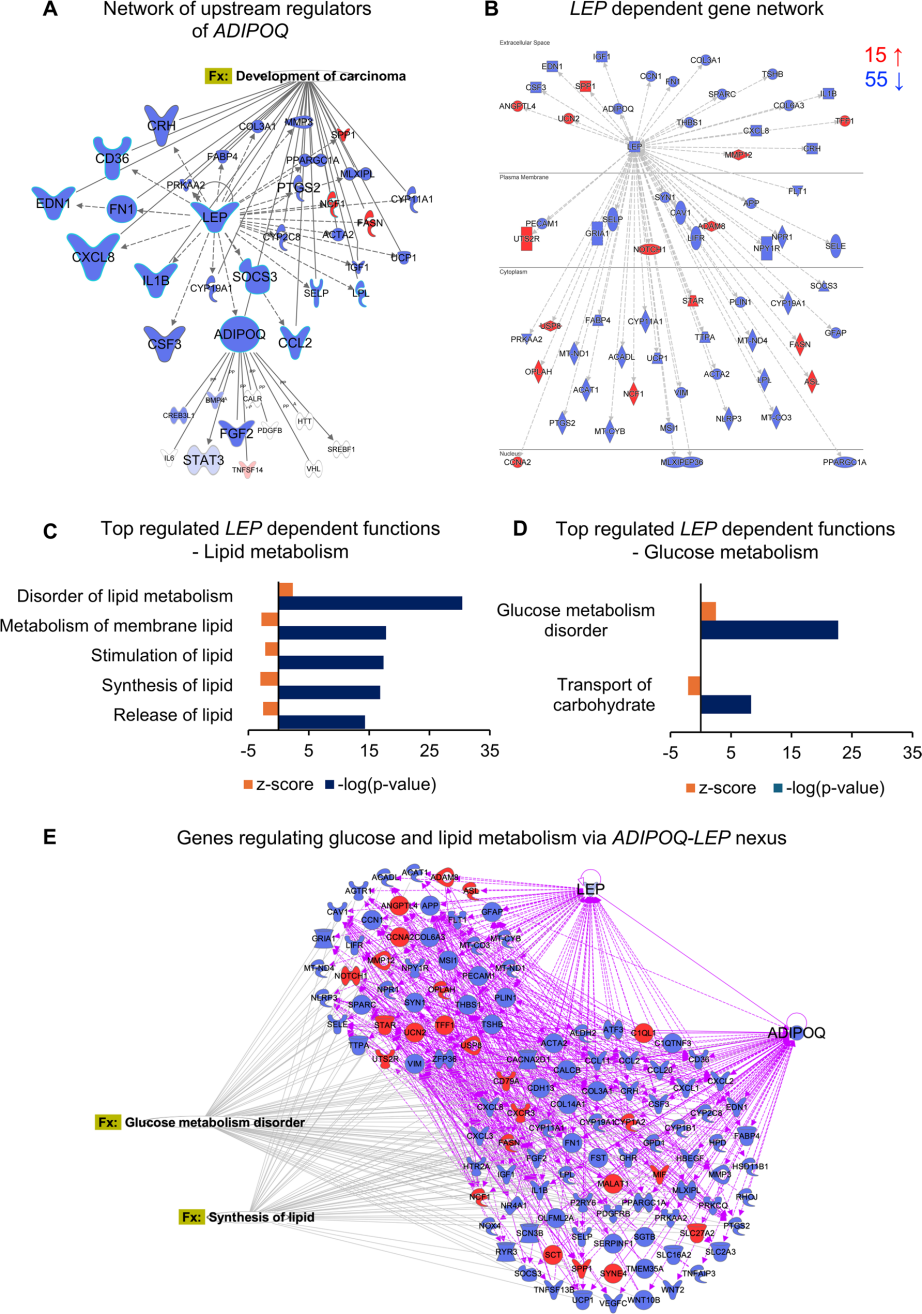
Regulation of *LEP* dependent genes in tumor biopsies from patients treated with alpha1-oleate. **A**) Network of ADIPOQ upstream regulators showing a predominance of inhibition (blue). LEP is identified as the most strongly downregulated gene (FC = -60). Complete list is shown in Supplementary Figure 3. **B**) Network of LEP dependent genes, 55 out of 70 genes are downregulated. **C**) Regulation of lipid metabolism functions by LEP gene network (orange = z-score, blue = -log(p-value)). Inhibition of lipid synthesis, release and stimulation of lipids and metabolism of membrane lipids is observed. **D**) Regulation of glucose metabolism functions by LEP gene network. Regulated functions include glucose metabolism disorder and transport of carbohydrate. **E**) Combined network of ADIPOQ and LEP related genes shows strong overlap and the predominance of downregulated genes

*LEP* was strongly downregulated (FC = -60) in treated tumor tissue biopsies and was identified as the top upstream regulator of *ADIPOQ*, suggesting a combined effect in alpha1-oleate treated patients (Fig. 3A, Supplementary Fig. 3). Additional upstream regulators of *ADIPOQ* included *PTGS2* and *CD36* predicting effects on lipid uptake, as well as pro-inflammatory cytokines *CRH*, *CXCL8*, *CCL11*, *IL1B*, *CCL2*, and *EDN1*, indicating downregulation of immune-driven metabolic signaling. The upstream regulators were also associated with carcinoma development, confirming the combined inhibition of cancer and metabolism.

The *LEP* dependent genes were identified as downregulated (55/70 significantly regulated genes, Fig. 3B). Network analysis further predicted inhibition of the *CD36* transmembrane receptor (FC = − 7.6), *LPL* (lipoprotein lipase, FC = − 4.3), *PLIN1* (Perilipin 1, FC = − 6.9), *ACADL* (Acyl-CoA dehydrogenase long chain, FC = − 2.7), and *ACAT1* (Acetyl-CoA acetyltransferase 1, FC = − 2.1), suggesting an overall effect on fatty acid uptake, storage, and synthesis. Functional analysis identified lipid synthesis, stimulation, release and metabolism as strongly inhibited, consistent with an inhibitory effect (Fig. 3C). Glucose metabolism disorder and carbohydrate transport functions were also strongly down-regulated (Fig. 3D).

The *LEP* and *ADIPOQ* networks were strongly interconnected, further supporting a convergent metabolic effect of *ADIPOQ* and *LEP* in alpha1-oleate treated tissues, with inhibition of metabolism compared to placebo (Fig. 3E and Supplementary Fig. 4). Shared regulated genes included *FABP4* (fatty acid binding protein 4, FC = -3.9), *FASN* (fatty acid synthase, FC = 2.98), *LPL* (FC = -4.34) and *CD36* (FC = − 7.6), associated with lipid synthesis and uptake. Similarly, *PPARGC1A*, (peroxisome proliferator-activated receptor gamma coactivator 1 alpha, FC = -5), *PRKAA2* (protein kinase AMP-activated catalytic subunit alpha 2, FC = -3.4) and *IGF1* (insulin growth factor 1, FC = -3) were strongly inhibited, predicting an association with energy and glucose metabolism as well as lipid metabolism in patients treated with alpha1-oleate.

The results identify metabolic genes as inhibited in cancer tissues from patients treated with alpha1-oleate and suggest that adiponectin and leptin are essential regulators of this response. The potent inhibition of both glucose and lipid metabolism may lead to starvation in cancer cells, ultimately reducing cancer cell growth and viability.

### Comparative analysis of biopsies from alpha1-oleate treated tumor tissue and benign tissue

**Fig. 4:**
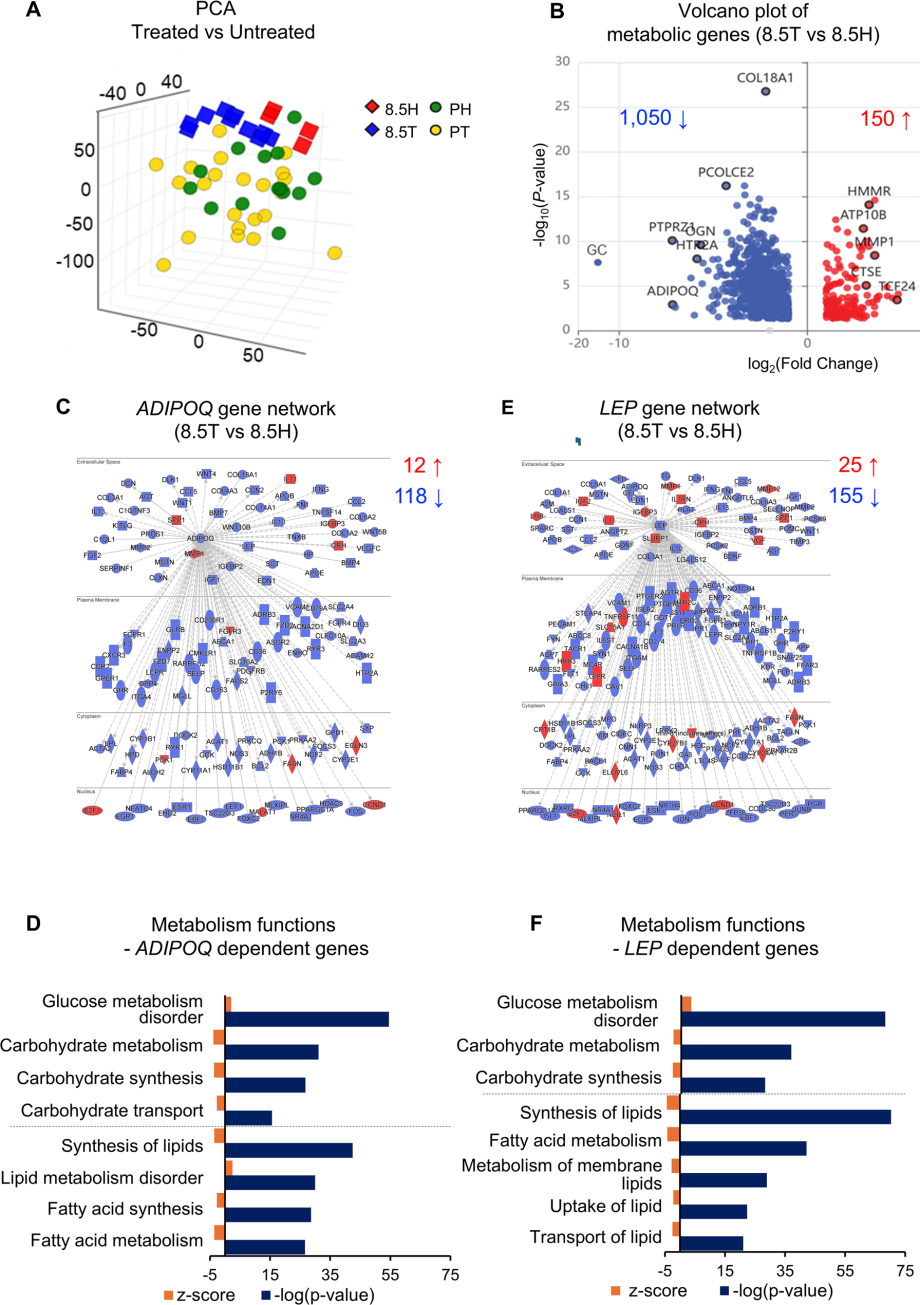
Gene expression profiling of the metabolic response to alpha1-oleate in biopsies obtained from tumor tissues compared to benign tissue areas. (**A**) Principal component analysis (PCA) of metabolic genes in RNA samples from tissues obtained at TURBT. Gene expression was compared between the tumors and areas with benign morphology and between tumors treated with 8.5 mM of alpha1-oleate or placebo. The X-axis represents Principal Component 1 (PC2), the Y-axis PC1 and the Z-axis PC3. The alpha1-treated tumors clustered at a distance from the placebo treated tumors, supporting the observed treatment effect on metabolism. The alpha1-oleate treated benign tissue biopsies clustered at a distance from the placebo control, closer to the treated tumors, suggesting a treatment effect, but less pronounced than for tumor tissue. (**B**) Direct comparison of metabolic gene expression in alpha1-oleate treated tissues versus benign tissues. Volcano plot of regulated metabolic genes illustrating the predominance of inhibition in alpha1-oleate treated tumor tissue (1050 downregulated and 150 upregulated genes, blue = downregulation, red = upregulation, FC > 2, *P* < 0.05). (**C**) Inhibition of the *ADIPOQ* related gene network in alpha1-oleate treated tumor tissue compared to benign tissue. (**D**) Inhibition of *ADIPOQ* dependent glucose and lipid metabolism functions regulated by *ADIPOQ* (orange = z-score, blue = -log(p-value))) compared to benign tissue. (**E**) Inhibition of the *LEP* related gene network in alpha1-oleate treated tumor tissue compared to benign tissue. (**F**) Inhibition of *LEP* dependent glucose and lipid metabolism in alpha1-oleate treated tumor tissue compared to benign tissue

Bladder cancer is known to have a field effect wherein “healthy” tissue likely still includes cancer cells or pre-cancerous cells. The metabolic response in alpha1-oleate treated tumors was therefore compared to biopsies from macroscopically benign bladder tissue areas, located at a distance from the tumor. Metabolism-associated genes were identified by RNA sequencing, and the overall distribution of metabolic genes was compared by principal component analysis, compared to placebo (PCA). Samples from alpha1-oleate treated patients were shown to cluster at distance from the placebo group (PCA in Fig. 4A), suggesting a treatment effect in both tumor tissue and benign tissue biopsies.

The metabolic response to alpha1-oleate treatment was directly compared between the treated tumor biopsies and the benign tissue biopsies. About 1200 genes were significantly regulated only in the tumor group (FC 2, *P* < 0.05), demonstrating a stronger effect of alpha1-oleate treatment on gene expression in tumor tissues than benign tissues. The distribution of genes specifically regulated in the tumor biopsies is shown in the Volcano plot in Fig. 4B. The majority of metabolism-associated genes were inhibited in the tumors (1050/1200, 87% of significantly regulated genes). Network analysis confirmed the inhibition of *ADIPOQ-* (118/130 genes) and *LEP-* (155/180 genes) dependent genes (Fig. 4C and E). Functional analysis identified glucose metabolism disorder and carbohydrate metabolism and synthesis genes as well as lipid synthesis genes, as strongly inhibited (Fig. 4D and F).

The results illustrate the potent effects of alpha1-oleate treatment on tumor tissue compared to benign areas of the bladder.

### Local metabolic effects of alpha1-oleate defined by sequencing of urine RNA

To further investigate the local effects on tumor cell metabolism, urine samples were collected within 2 h of alpha1-oleate instillation and urine RNA was subjected to genome wide transcriptomic analysis. Samples collected before and after treatment were compared.

**Fig. 5:**
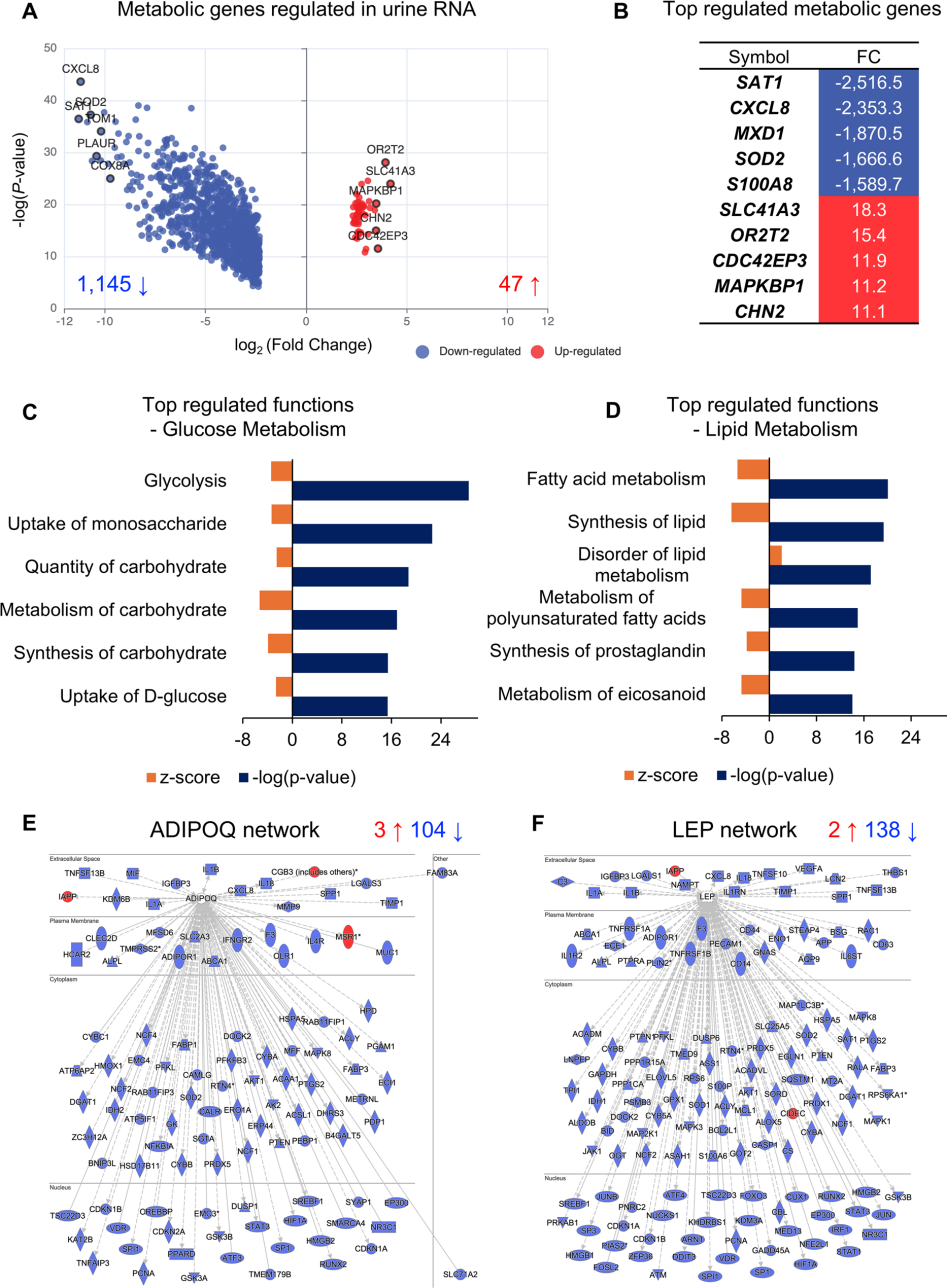
Gene expression profiling of the metabolic response to intravesical alpha1-oleate treatment in cells shed into the urine. (**A**) Volcano plot of regulated metabolic genes illustrating the predominance of inhibition after alpha1-oleate treatment compared to pre-inoculation samples (1145 downregulated and 47 upregulated genes) (FC ≥ 5 and P < 0.05, red = upregulation, blue = downregulation). (**B**) Top differentially expressed metabolic genes in post-instillation urine samples in alpha1-oleate patients compared to pre-instillation samples. (**C**) Regulation of glucose metabolism functions by significantly expressed metabolism genes in urine RNA (orange = z-score, blue = -log(p-value)). Glycolysis, uptake, quantity, metabolism and synthesis of glucose are inhibited. **D**). Regulation of lipid metabolism functions by significantly expressed metabolism genes in urine RNA. Fatty acid metabolism, synthesis of lipid and prostaglandin are inhibited. **E**) Network of ADIPOQ related genes, 104 out of 107 genes are inhibited. **F**) Network of LEP related genes, 138 out of 140 genes are inhibited

The inhibition of metabolic genes is illustrated by the volcano plot in Fig. 5A, (1145/1192 regulated genes, 96% inhibition, high cut off FC > 5, *P* < 0.05). The top downregulated gene *SAT1* (Spermidine/Spermine N^1^ Acetyltransferase 1) is a known biomarker for bladder cancer, which regulates polyamine synthesis. *SAT1* is also a positive regulator of insulin sensitivity and lipid peroxidation (Fig. 5B). Functional analysis of glucose metabolism identified glycolysis, glucose uptake and metabolism as inhibited, as well as lipid metabolism and prostaglandin synthesis (Fig. 5C-D). Adiponectin and Leptin were not among the top regulated genes, but *ADIPOQ* and *LEP* networks showed a predominance of inhibition, including the adiponectin receptor R1. The solute carrier *SLC41A* was identified as the top upregulated metabolic gene, involved in Mg^2+^ homeostasis.

The results suggest a rapid change in metabolic gene expression and metabolic functions in treated tumor cells, compared to samples obtained before treatment.

### Adipokine levels in clinical samples from alpha1-oleate treated patients compared to placebo

The metabolic response to alpha1-oleate treatment was first evaluated by quantifying adipokine levels in urine using a customized Luminex human adipokine panel. Urine samples were collected at visit 1, before treatment (Pre V1) and pre- and post-treatment, at each visit (Post V1-V6). No change in adipokine levels was detected in the placebo group for any of the measured proteins, indicating that the intravesical instillation procedure itself did not affect urinary adipokine concentrations.

**Fig. 6:**
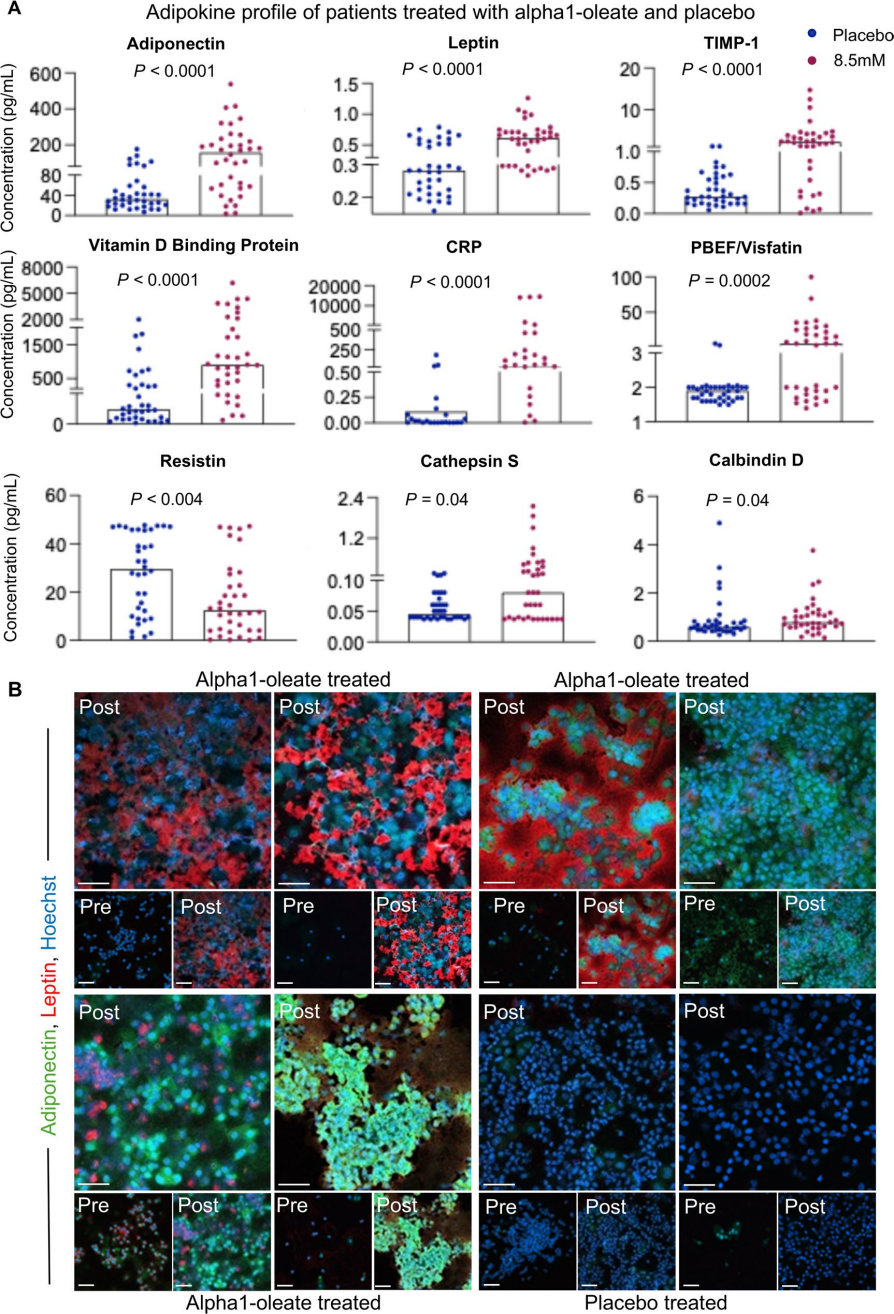
Adipokine response to alpha1-oleate instillation quantified in urine and shed tumor cells. **A**) Urine samples were collected before and after intravesical instillation of alpha1-oleate or placebo (*n* = 6 patients per group, 6 visits per patient). Adipokines were quantified using the Luminex Human Metabolic Panel (16 proteins). Alpha1-oleate treatment increased urinary levels of Adiponectin, Leptin, TIMP-1, Vitamin D Binding Protein, PBEF/Visfatin, and Cathepsin S (Mann Whitney *U*-test), compared to placebo. **B**) Cytospin preparations of shed cells in urine were immunostained for adiponectin (green) and leptin (red). Three alpha1-oleate–treated patients and one patient in the placebo group are shown (visits 3 and 6, pre- and post-instillation). Representative images from alpha1-oleate-treated patients illustrates higher adiponectin or leptin staining post-instillation. Data for additional three alpha1-oleate treated patients and five patients in the placebo group are shown in Supplementary Figure 8 and 9 respectively. Scale bar, 50 μm

A significant adipokine response was detected in the alpha1-oleate treatment group, compared to placebo (Fig. 6). Increased urine adipokine levels in the post-alpha1-oleate instillation samples were observed for Adiponectin, Leptin, Vitamin D, TIMP-1, PBEF/Visfatin and Cathepsin S (Fig. 6A). In contrast, the urine levels of Insulin, TREM-1, IGFBP-rp1/IGFBP-7, CXCL2, Myeloperoxidase, DPPIV/CD26, or M-CSF remained unchanged (Supplementary Fig. 5). Resistin levels were lower in the alpha1-oleate treatment group than in the placebo group.

The response kinetics are shown in Supplementary Fig. 6. A rapid response was detected in the alpha-oleate treated patients compared to the placebo group for adiponectin, leptin, Vitamin D, TIMP-1 and PBEF/Visfatin. Additionally, the response was maintained for Adiponectin, Leptin, Vitamin D and TIMP-1, compared to pre-inoculation samples, suggesting both an acute and sustained response (Supplementary Fig. 7).

### Staining for adiponectin in cells shed into the urine

Alpha1-oleate triggers rapid tumor cell detachment, within two hours of intravesical instillation [20]. The number of cells increases from baseline levels up to several hundred thousand or millions of cells per mL of urine, offering a useful source of cells to investigate the tumor response to treatment. To further investigate the tissue origin of the adipokine response, matched pre-treatment and post-treatment urine samples were collected from a subset of patients and the response was compared between the treatment and placebo groups at visits 3 and 6. Higher cellular adiponectin levels in shed cells and tissue fragments were detected by immunostaining, post treatment than before treatment, consistent with the increase in urine adipokine levels (Fig. 6B). The adiponectin staining intensity was also higher in the post-treatment samples from alpha1-oleate treated patients than in the placebo group, but with heterogeneity of staining (Fig. 6B, Supplementary Figs. 8 and 9). The leptin staining was detected in three patients treated with alpha1-oleate and one patient in the placebo group (Fig. 6B and Supplementary Fig. 9). Lower urine RNA levels for adiponectin and leptin gene networks were detected in the same urine samples, where shed tumor cells contained high levels of adiponectin protein, apparently activated in response to treatment. Urine samples obtained before treatment and samples from the placebo group did not show increased protein levels, suggesting that both end points are treatment related.

## Discussion

This study identifies the alpha1-oleate complex as an efficient inhibitor of tumor metabolism in patients with non-muscle invasive bladder cancer. The study further identified convergent adiponectin and leptin dependent gene networks, whose inhibition was associated with rapid and potent effects on the metabolic machinery of the tumor. The effects were further enhanced by interactions with other top-regulated metabolic gene networks, providing a molecular basis for the broad inhibition of tumor metabolism that was detected. Functional analysis confirmed effects on glucose and lipid metabolism accompanying the inhibition of cancer-related gene expression previously observed in these tumors [20]. Increased adipokine levels in the urine of alpha1-oleate treated patients and in shed tumor cells confirmed the direct effect of alpha1-oleate on tumor tissue. The results suggest a new way of targeting tumor metabolism, in patients with bladder cancer and other tumors that can be accessed by local treatment.

Extensive metabolic reprogramming is a characteristic of bladder cancer, suggesting that metabolism might be a promising target for therapeutic intervention [5, 23, 24]. The expression of *ADIPOQ*, *LEP* and their receptors correlates with tumor recurrence and progression, and high serum adiponectin levels were identified as an independent predictor of bladder cancer progression [25]. The upregulation of *PAQR4* (ADIPOQ Receptor Family Member 4), was proposed to promote bladder cancer progression by upregulating the PI3K/AKT pathway [26] and leptin dysregulation activates the JAK/STAT, PI3K/Akt/mTOR, MAPK/ERK and RAS pathways, promoting proliferation, survival, migration and angiogenesis in various cancers [27]. In this study, alpha1-oleate was shown to inhibit the expression of *ADIPOQ*, *LEP* and their receptors as well as networks involving JAK/STAT, PI3K/Akt/mTOR, MAPK/ERK and RAS, suggesting efficient targeting of malignant bladder cancer cells that have undergone metabolic reprogramming. The inhibitory effects were more pronounced in tumor tissue than in biopsies from morphologically benign tissue, consistent with a much lower number of malignant cells predicted to reside in bladder areas without macroscopically detectable tumors. The potent response in the tumor and weaker response in benign tissue is encouraging, as it suggests that alpha1-oleate also targets malignant cells that are not detected by the naked eye, at cystoscopy.

While the study confirms *ADIPOQ*, *LEP* and their downstream pathways as core regulatory targets of alpha1-oleate, the molecular mechanism by which this complex regulates the expression of these two key adipokines are not addressed. Alpha1-oleate is formed by the 39-mer N-terminal alpha1 peptide of human alpha-lactalbumin (residues 20–59) and three to five oleic acid molecules. The peptide has been defined by NMR as partially unfolded, surrounding a hydrophobic oleic acid core in the complex [1]. Interesting molecular interactions of alpha1-oleate, relevant to this study, were recently observed in silico, using AlphaFold 3. The alpha1-oleate complex was predicted to interact with a surface exposed domain of the adiponectin receptor AdipoR2 and with a previously identified oleic acid binding cavity in the transmembrane domain of this receptor [28]. Binding was confirmed by surface plasmon resonance, using Biacore technology [22]. Furthermore, in silico modeling detected an interaction with the Ig-like domain of the leptin receptor, an interaction, also confirmed at the peptide level (unpublished observation). The results suggest that alpha1-oleate may recognize and bind to adiponectin and leptin receptors, providing insights into some of the metabolic effects observed in the tumors.

Cisplatin resistance was recently linked to metabolic reprogramming by anti-tumor therapy, as a mechanism supporting tumor cell survival under therapeutic stress [29–32]. Affected metabolic functions in surviving cells included enhanced glycolysis, lipid metabolism, mitochondrial adaptation and redox balance. The broad inhibition of glucose and lipid metabolism in alpha1-oleate treated tumors, together with the suppression of *ADIPOQ*- and *LEP*-dependent signaling networks suggests an ‘’inverse metabolic reprogramming,’’ which would not be expected to promote cell proliferation or enhance malignancy but to inhibit the hyperactive metabolic environment of the tumor and promote a return towards a healthier phenotype. Cells repeatedly exposed to alpha1-oleate show no evidence of resistance, as lethal concentrations of the drug still activate programmed cell death and kill the cells. The present study shows that the activation of cell death, which is accompanied by inhibition of the oncogene driven tumor microenvironment genes, predicted to inhibit metastasis, neovascularization, proliferation and migration [20], also involves the metabolic machinery.

In contrast to studies focusing on serum adiponectin and leptin, which primarily investigate systemic metabolic status, our study demonstrated that *ADIPOQ* and *LEP* are actively expressed within bladder cancer tissue itself, together with coordinated activation of downstream metabolic signaling pathways. This supports a model in which adipokines function as locally expressed regulators of tumor metabolism and survival. Recent studies have identified a seven-gene metabolic signature in bladder cancer (Metab-GS) as associated with progression toward a more aggressive, muscle-invasive phenotype or increased invasive potential of less-aggressive tumors, comprising *ALDH1B1/L2* (Aldehyde Dehydrogenase 1 Family Member B1/L2), *CHSY1* and *CSGALNACT2* (Chondroitin Sulfate Synthase 1 and N-Acetyl-galactosaminyl-transferase 2), *GPX8* (Glutathione Peroxidase 8), *FBP1* (Fructose-Bisphosphatase 1), and *HPGD* (15-Hydroxyprostaglandin Dehydrogenase) [23]. Other metabolic genes associated with bladder cancer metabolism are mTORC1 signaling that enhances glycolysis and *de novo* lipid and cholesterol biosynthesis [33] in early-stage NMIBC tumors of the GS2 genomic subtype, *ACAT1* (Acetyl-CoA acetyltransferase 1) and *GLUT1/3* (Glucose Transporter Type 1/3), which regulate glucose metabolism are upregulated in bladder cancer [34, 35]. Activated glycolysis-related enzymes and transporters further include *SRC-3* (Nuclear receptor coactivator 3) [36], *LDHA/B* (Lactate dehydrogenase A/B) [37], *HK1/2* (hexokinase 1/2), *PKM* (pyruvate kinase type M) [38], and *HIF-1α* (hypoxia-inducible factor 1-alpha) [24, 34, 39]. Alpha1-oleate treatment was shown in this study to inhibit several of the identified signature genes, including glucose transporters *SLC2A3* (*GLUT3)* and *ACAT1* as well as *ALDH1L2.* In addition, *HIF-1α* signaling was strongly inhibited (z-score = -3.1), consistent with the early siRNA-based screen identifying *HIF-1α* as a determinant of HAMLET sensitivity in tumor cells [40]. The results identify alpha1-oleate as a broad metabolic regulator and highlight the therapeutic potential of the ‘HAMLET family’ of molecules in treating cancer as well as metabolic disease.

## Supplementary Information

Below is the link to the electronic supplementary material.

Supplementary Material 1

## Data Availability

The RNA sequencing data produced in this study have been made publicly available through the Gene Expression Omnibus (GEO) repository under accession number GSE172112 and are also accessible at the BioProject database with the accession number PRJNA1119936.
