# Supplementary material for: Identification of alpha1-oleate as a potent regulator of adipokine-dependent metabolism, in bladder cancer tissue
Source: Cancer Metab. 2026 Jun 23;14:21. doi: 10.1186/s40170-026-00445-2 (PMC13292342; doi:10.1186/s40170-026-00445-2)
Supplement: Supplementary file 1 — Supplementary Material 1 [file 40170_2026_445_MOESM1_ESM.pptx]

## Slide 1
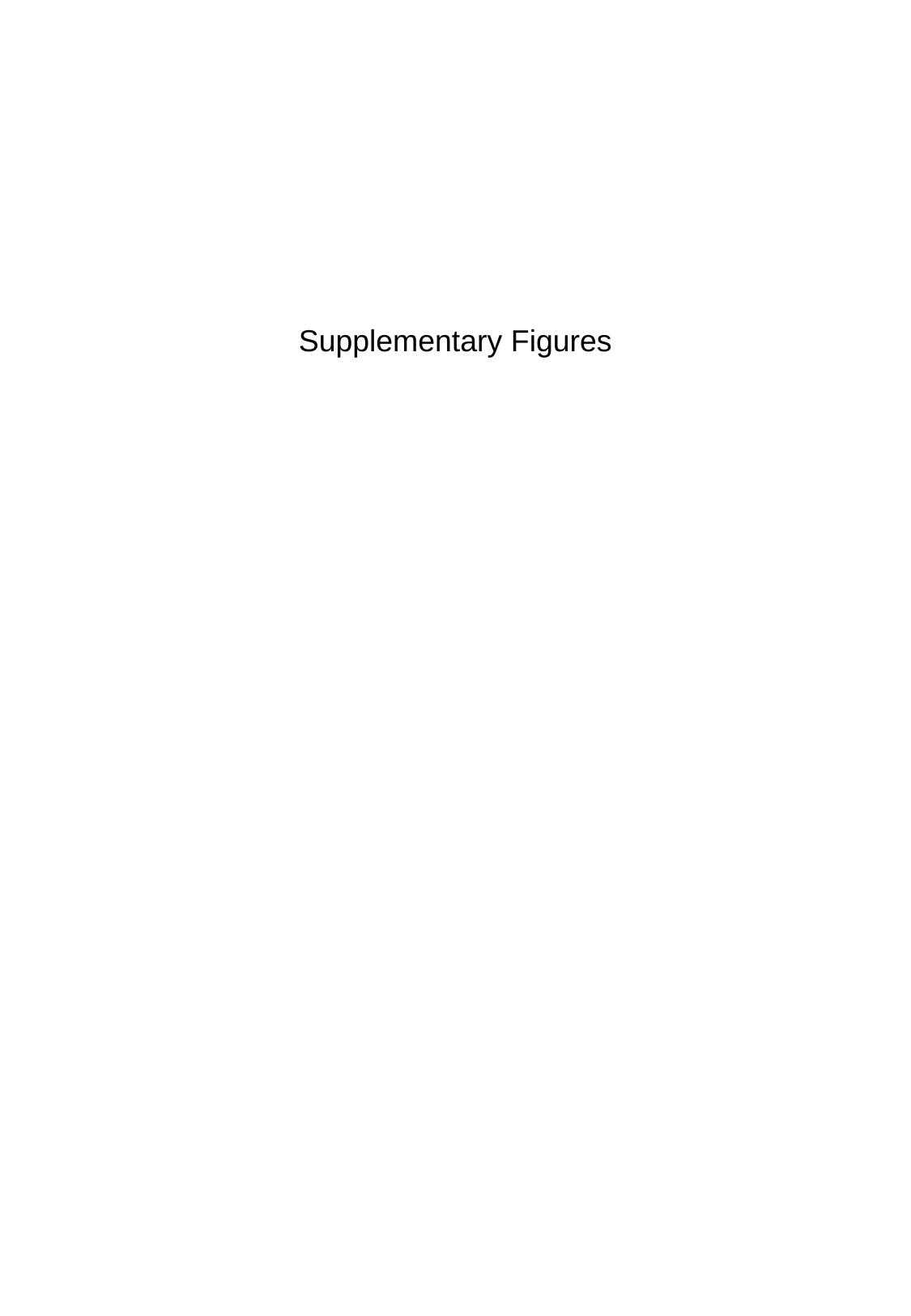

Supplementary Figures

## Slide 2
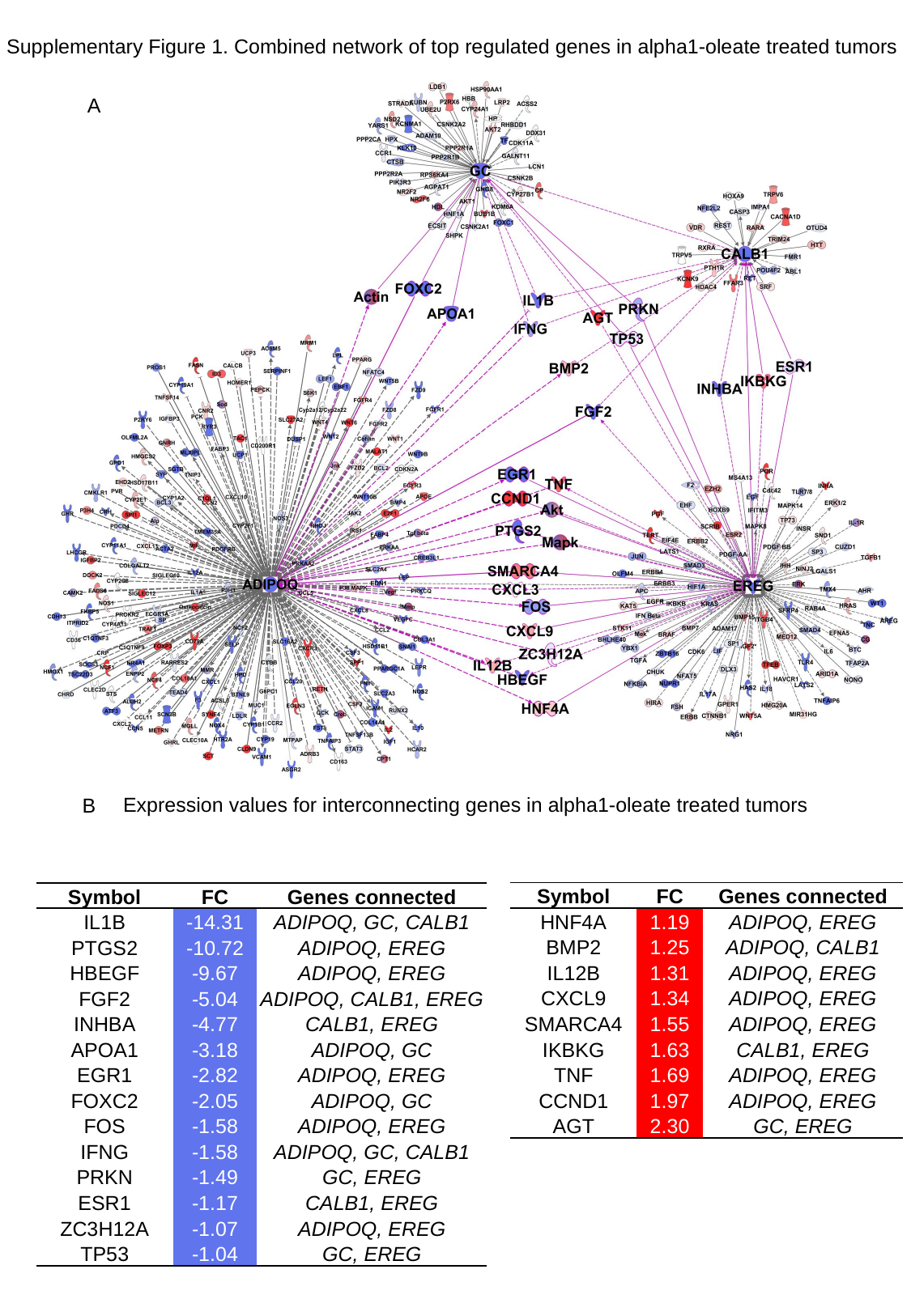

Supplementary Figure 1. Combined network of top regulated genes in alpha1-oleate treated tumors
A
Expression values for interconnecting genes in alpha1-oleate treated tumors
B
| Symbol | FC | Genes connected |
| --- | --- | --- |
| HNF4A | 1.19 | ADIPOQ, EREG |
| BMP2 | 1.25 | ADIPOQ, CALB1 |
| IL12B | 1.31 | ADIPOQ, EREG |
| CXCL9 | 1.34 | ADIPOQ, EREG |
| SMARCA4 | 1.55 | ADIPOQ, EREG |
| IKBKG | 1.63 | CALB1, EREG |
| TNF | 1.69 | ADIPOQ, EREG |
| CCND1 | 1.97 | ADIPOQ, EREG |
| AGT | 2.30 | GC, EREG |
| Symbol | FC | Genes connected |
| --- | --- | --- |
| IL1B | -14.31 | ADIPOQ, GC, CALB1 |
| PTGS2 | -10.72 | ADIPOQ, EREG |
| HBEGF | -9.67 | ADIPOQ, EREG |
| FGF2 | -5.04 | ADIPOQ, CALB1, EREG |
| INHBA | -4.77 | CALB1, EREG |
| APOA1 | -3.18 | ADIPOQ, GC |
| EGR1 | -2.82 | ADIPOQ, EREG |
| FOXC2 | -2.05 | ADIPOQ, GC |
| FOS | -1.58 | ADIPOQ, EREG |
| IFNG | -1.58 | ADIPOQ, GC, CALB1 |
| PRKN | -1.49 | GC, EREG |
| ESR1 | -1.17 | CALB1, EREG |
| ZC3H12A | -1.07 | ADIPOQ, EREG |
| TP53 | -1.04 | GC, EREG |

## Slide 3
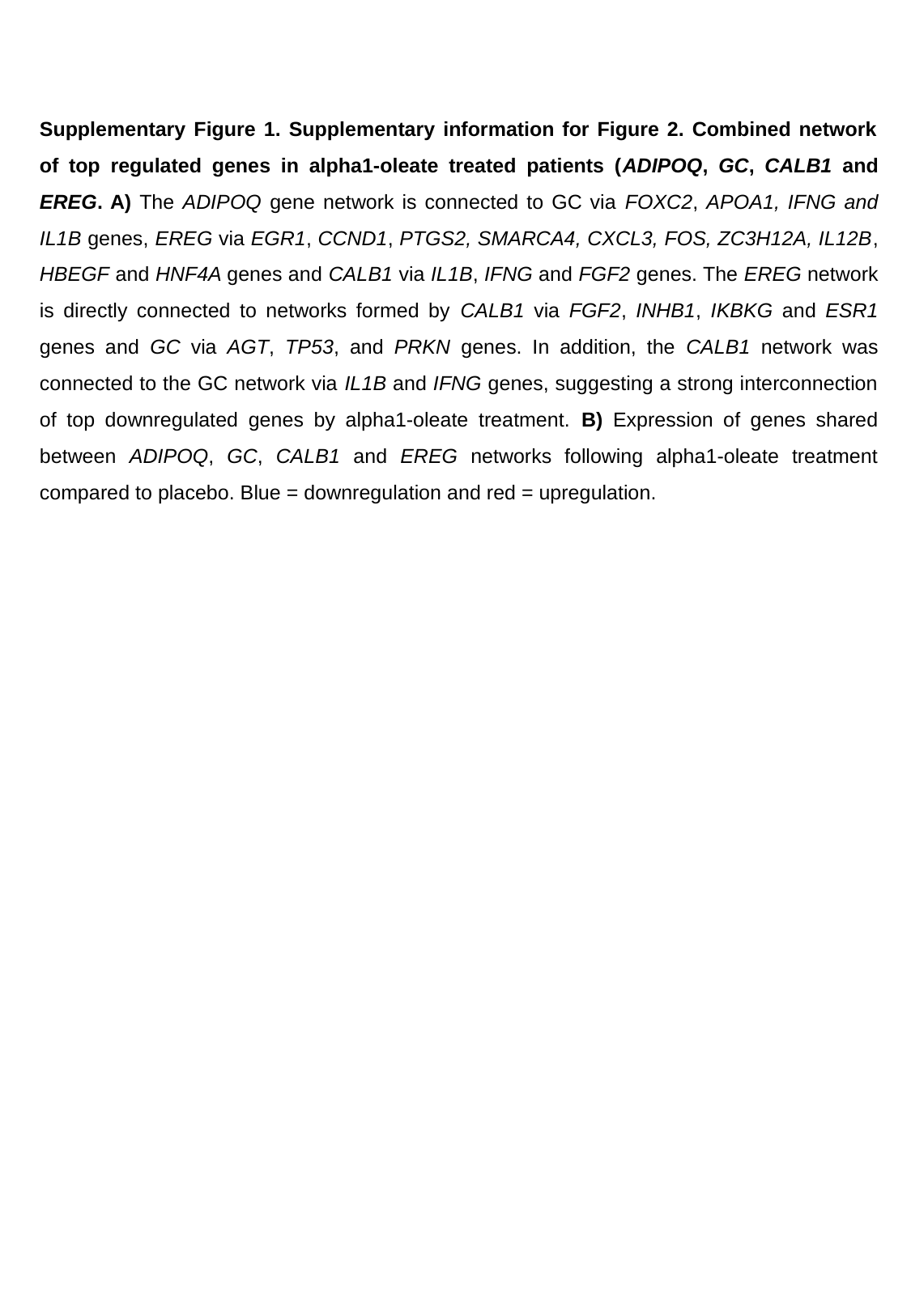

Supplementary Figure 1. Supplementary information for Figure 2. Combined network of top regulated genes in alpha1-oleate treated patients (ADIPOQ, GC, CALB1 and EREG. A) The ADIPOQ gene network is connected to GC via FOXC2, APOA1, IFNG and IL1B genes, EREG via EGR1, CCND1, PTGS2, SMARCA4, CXCL3, FOS, ZC3H12A, IL12B, HBEGF and HNF4A genes and CALB1 via IL1B, IFNG and FGF2 genes. The EREG network is directly connected to networks formed by CALB1 via FGF2, INHB1, IKBKG and ESR1 genes and GC via AGT, TP53, and PRKN genes. In addition, the CALB1 network was connected to the GC network via IL1B and IFNG genes, suggesting a strong interconnection of top downregulated genes by alpha1-oleate treatment. B) Expression of genes shared between ADIPOQ, GC, CALB1 and EREG networks following alpha1-oleate treatment compared to placebo. Blue = downregulation and red = upregulation.

## Slide 4
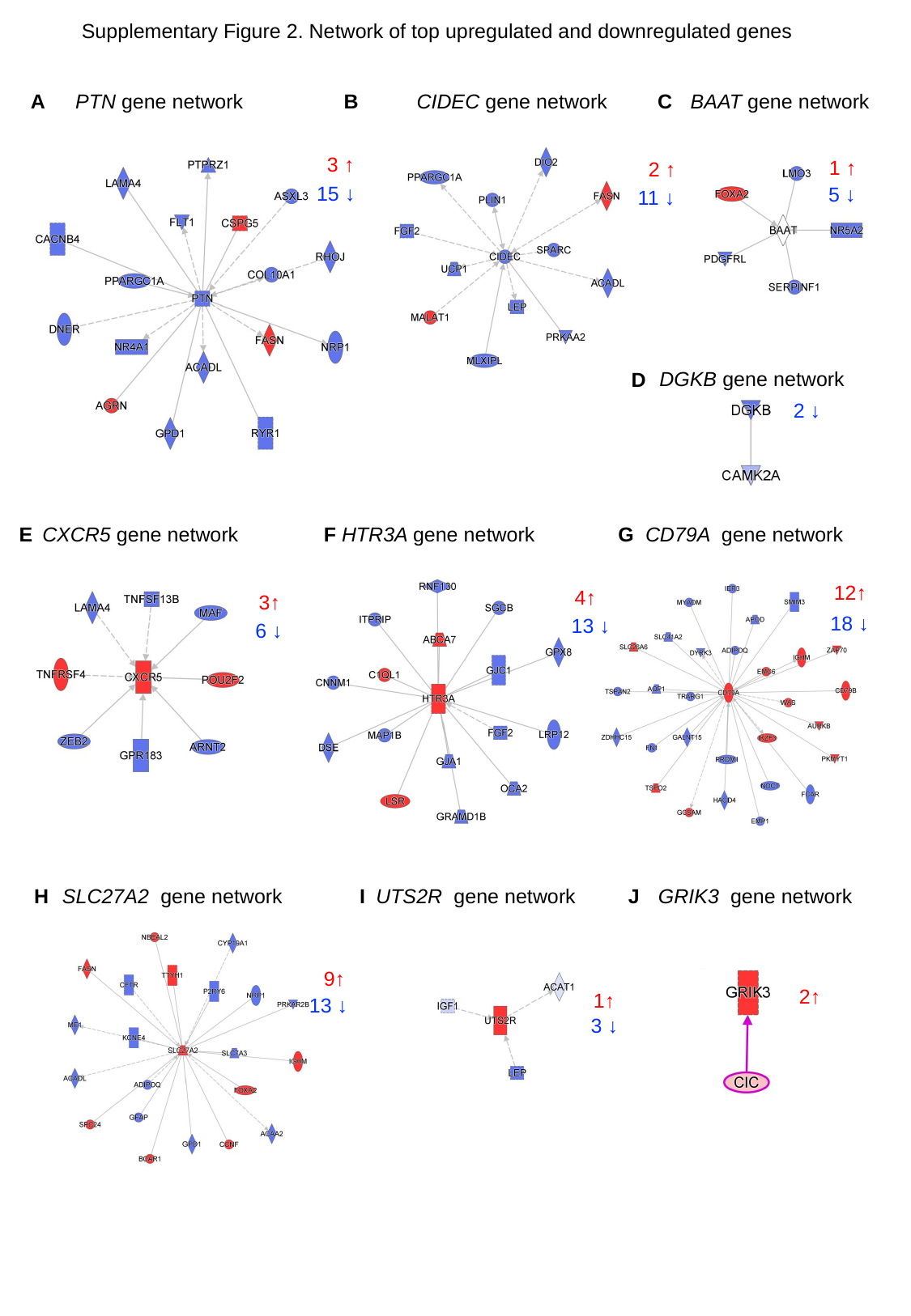

Supplementary Figure 2. Network of top upregulated and downregulated genes
PTN gene network
A
B
CIDEC gene network
BAAT gene network
C
3 ↑
1 ↑
2 ↑
15 ↓
5 ↓
11 ↓
DGKB gene network
D
2 ↓
CXCR5 gene network
E
HTR3A gene network
F
CD79A gene network
G
12↑
4↑
3↑
18 ↓
13 ↓
6 ↓
SLC27A2 gene network
H
UTS2R gene network
I
GRIK3 gene network
J
9↑
2↑
1↑
13 ↓
3 ↓

## Slide 5
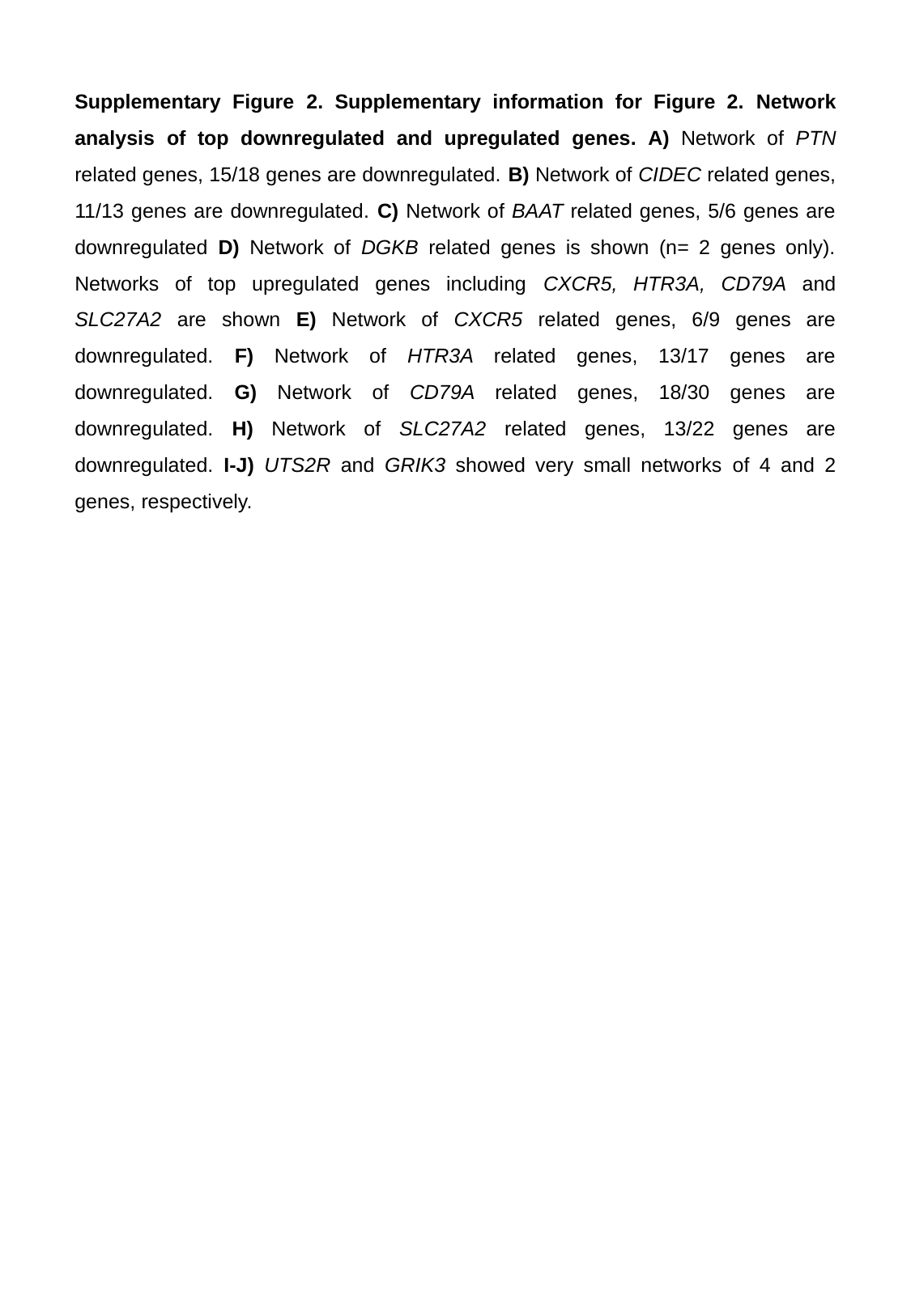

Supplementary Figure 2. Supplementary information for Figure 2. Network analysis of top downregulated and upregulated genes. A) Network of PTN related genes, 15/18 genes are downregulated. B) Network of CIDEC related genes, 11/13 genes are downregulated. C) Network of BAAT related genes, 5/6 genes are downregulated D) Network of DGKB related genes is shown (n= 2 genes only). Networks of top upregulated genes including CXCR5, HTR3A, CD79A and SLC27A2 are shown E) Network of CXCR5 related genes, 6/9 genes are downregulated. F) Network of HTR3A related genes, 13/17 genes are downregulated. G) Network of CD79A related genes, 18/30 genes are downregulated. H) Network of SLC27A2 related genes, 13/22 genes are downregulated. I-J) UTS2R and GRIK3 showed very small networks of 4 and 2 genes, respectively.

## Slide 6
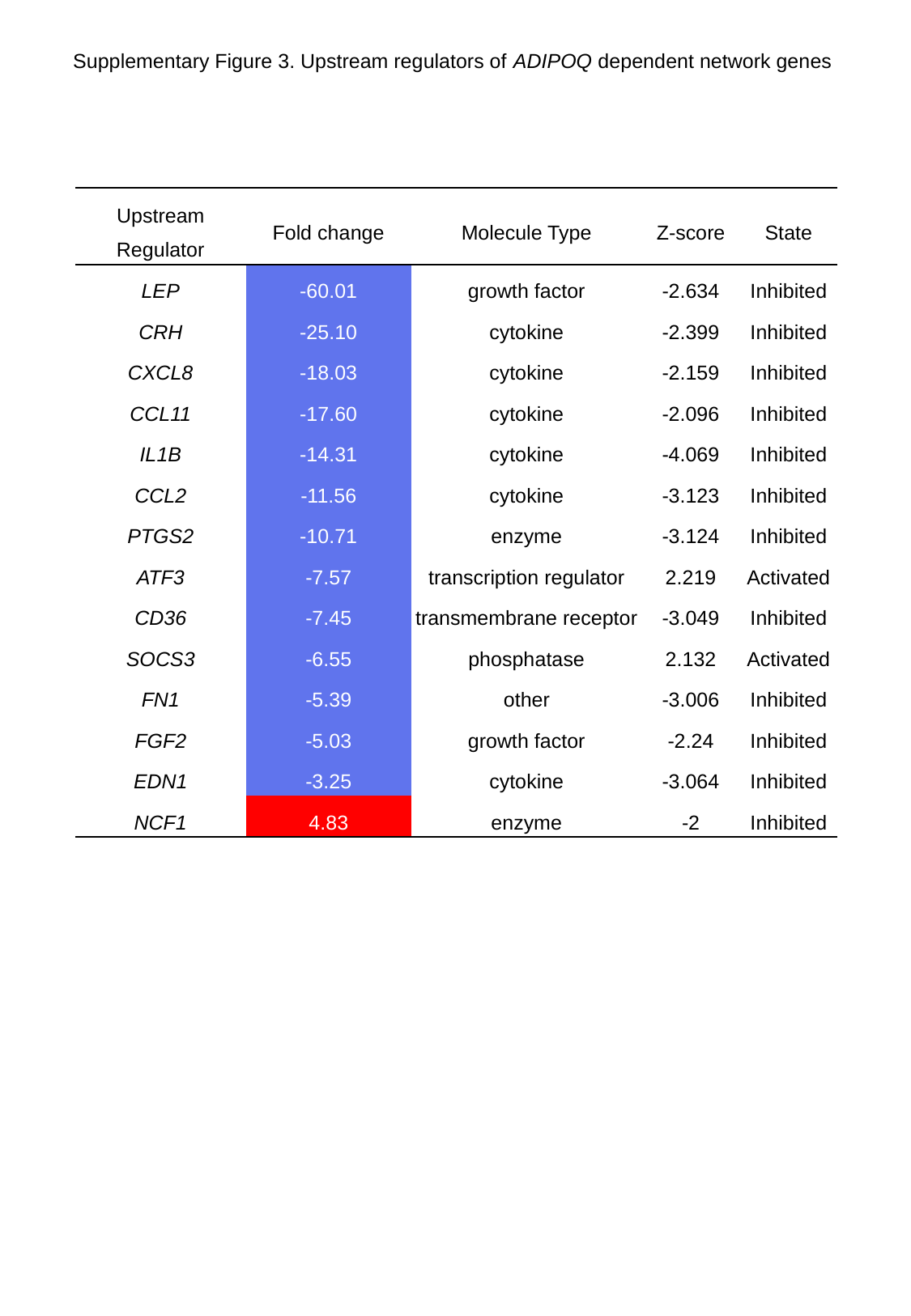

Supplementary Figure 3. Upstream regulators of ADIPOQ dependent network genes
| Upstream Regulator | Fold change | Molecule Type | Z-score | State |
| --- | --- | --- | --- | --- |
| LEP | -60.01 | growth factor | -2.634 | Inhibited |
| CRH | -25.10 | cytokine | -2.399 | Inhibited |
| CXCL8 | -18.03 | cytokine | -2.159 | Inhibited |
| CCL11 | -17.60 | cytokine | -2.096 | Inhibited |
| IL1B | -14.31 | cytokine | -4.069 | Inhibited |
| CCL2 | -11.56 | cytokine | -3.123 | Inhibited |
| PTGS2 | -10.71 | enzyme | -3.124 | Inhibited |
| ATF3 | -7.57 | transcription regulator | 2.219 | Activated |
| CD36 | -7.45 | transmembrane receptor | -3.049 | Inhibited |
| SOCS3 | -6.55 | phosphatase | 2.132 | Activated |
| FN1 | -5.39 | other | -3.006 | Inhibited |
| FGF2 | -5.03 | growth factor | -2.24 | Inhibited |
| EDN1 | -3.25 | cytokine | -3.064 | Inhibited |
| NCF1 | 4.83 | enzyme | -2 | Inhibited |

## Slide 7
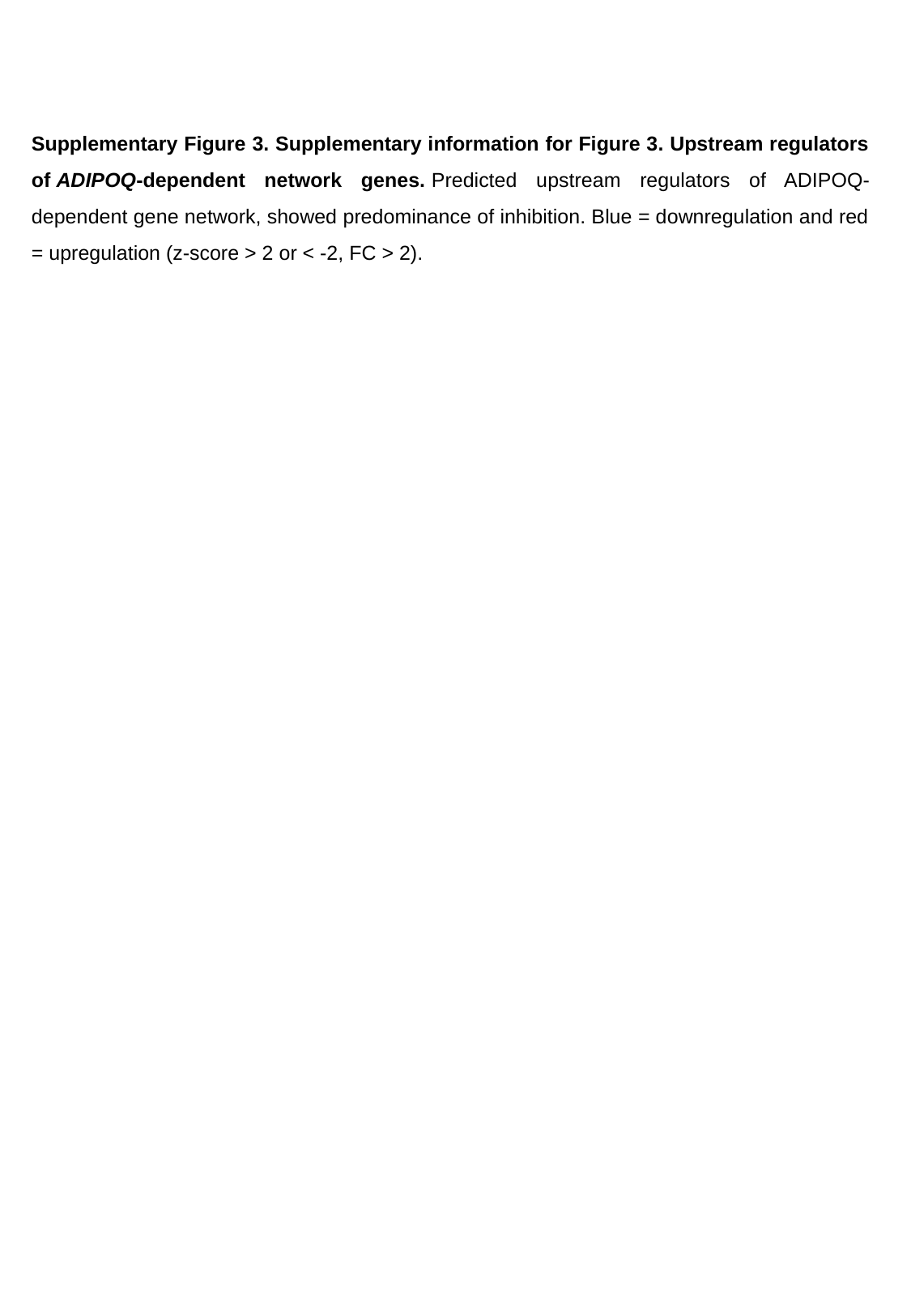

Supplementary Figure 3. Supplementary information for Figure 3. Upstream regulators of ADIPOQ-dependent network genes. Predicted upstream regulators of ADIPOQ-dependent gene network, showed predominance of inhibition. Blue = downregulation and red = upregulation (z-score > 2 or < -2, FC > 2).

## Slide 8
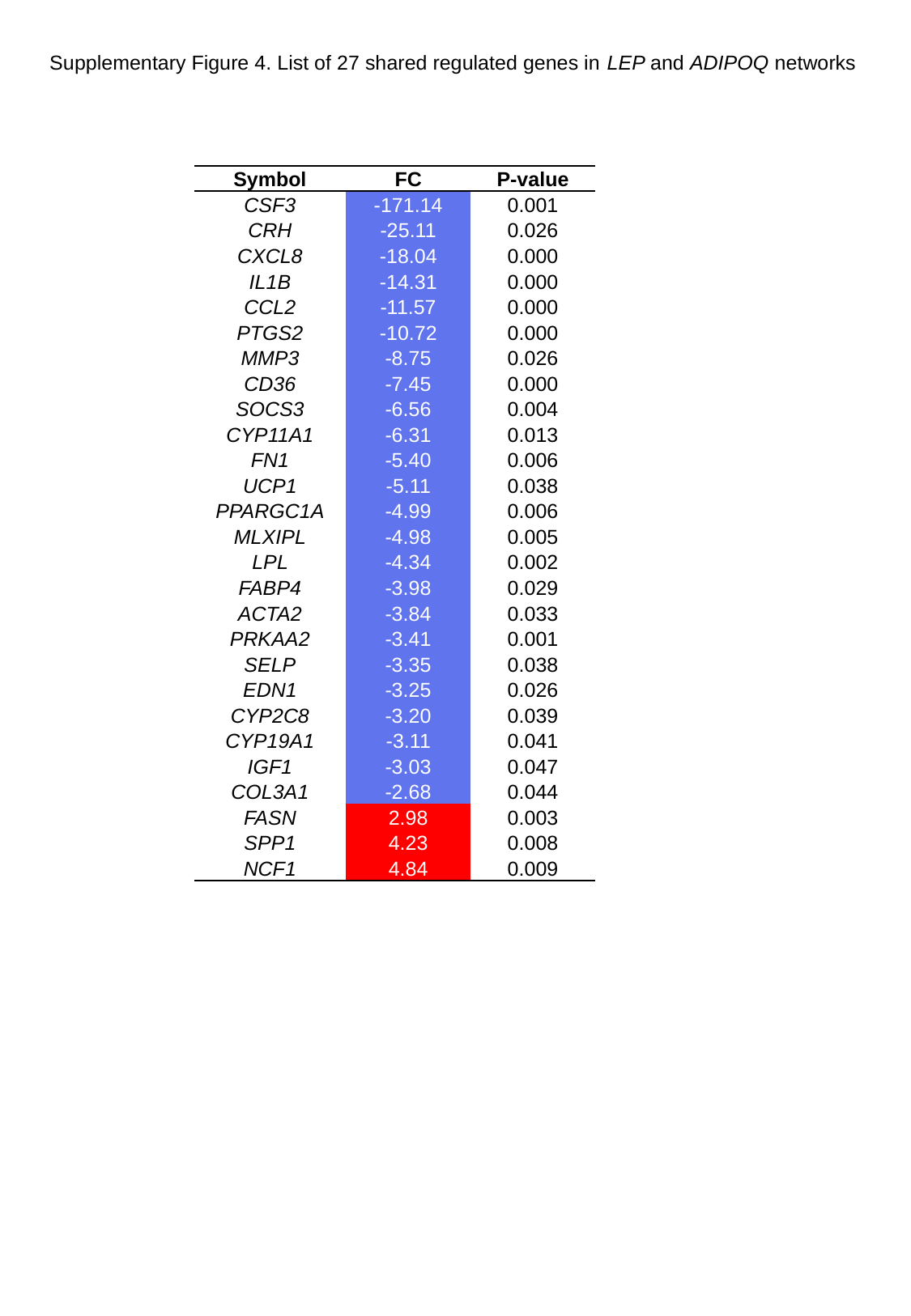

Supplementary Figure 4. List of 27 shared regulated genes in LEP and ADIPOQ networks
| Symbol | FC | P-value |
| --- | --- | --- |
| CSF3 | -171.14 | 0.001 |
| CRH | -25.11 | 0.026 |
| CXCL8 | -18.04 | 0.000 |
| IL1B | -14.31 | 0.000 |
| CCL2 | -11.57 | 0.000 |
| PTGS2 | -10.72 | 0.000 |
| MMP3 | -8.75 | 0.026 |
| CD36 | -7.45 | 0.000 |
| SOCS3 | -6.56 | 0.004 |
| CYP11A1 | -6.31 | 0.013 |
| FN1 | -5.40 | 0.006 |
| UCP1 | -5.11 | 0.038 |
| PPARGC1A | -4.99 | 0.006 |
| MLXIPL | -4.98 | 0.005 |
| LPL | -4.34 | 0.002 |
| FABP4 | -3.98 | 0.029 |
| ACTA2 | -3.84 | 0.033 |
| PRKAA2 | -3.41 | 0.001 |
| SELP | -3.35 | 0.038 |
| EDN1 | -3.25 | 0.026 |
| CYP2C8 | -3.20 | 0.039 |
| CYP19A1 | -3.11 | 0.041 |
| IGF1 | -3.03 | 0.047 |
| COL3A1 | -2.68 | 0.044 |
| FASN | 2.98 | 0.003 |
| SPP1 | 4.23 | 0.008 |
| NCF1 | 4.84 | 0.009 |

## Slide 9
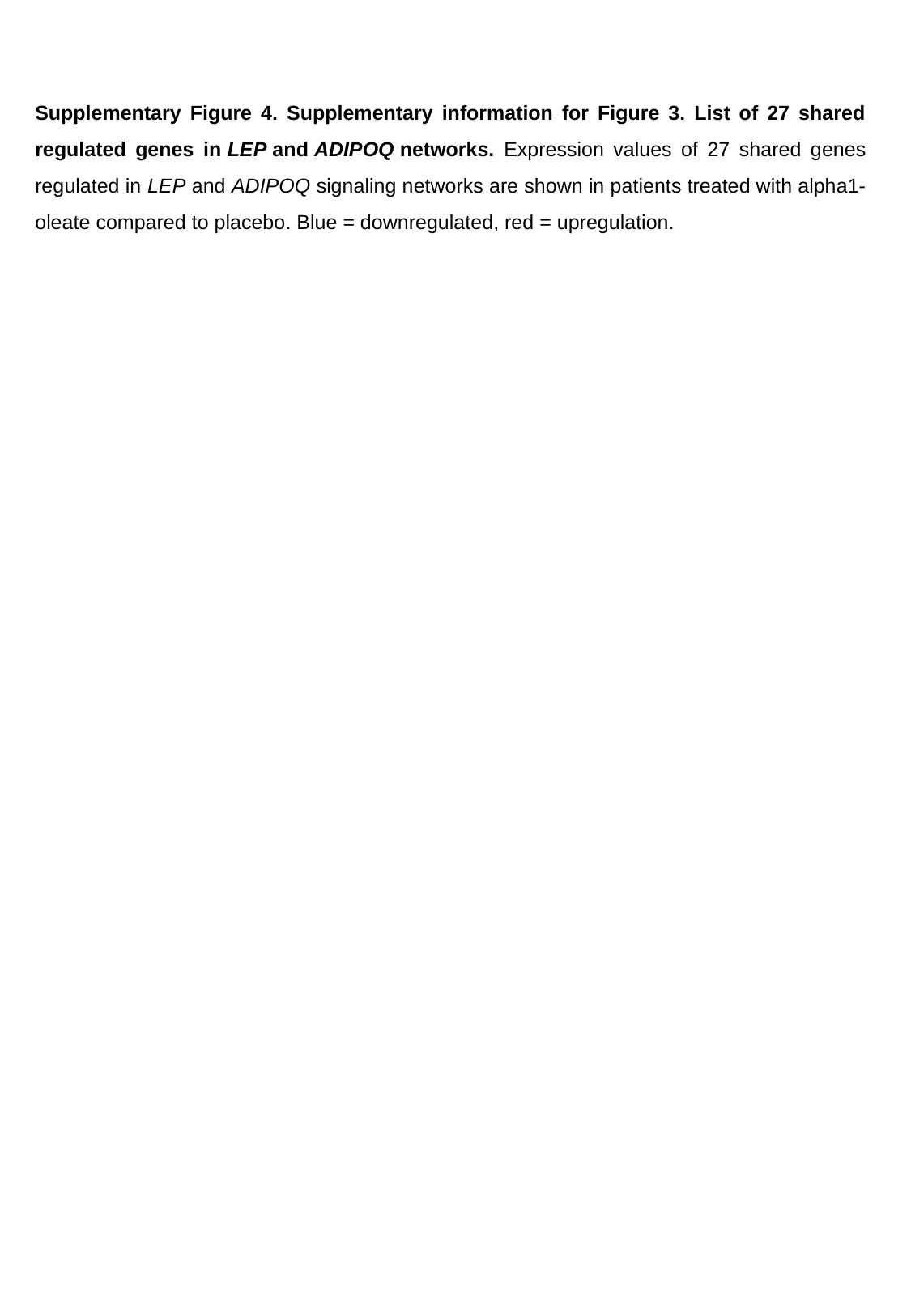

Supplementary Figure 4. Supplementary information for Figure 3. List of 27 shared regulated genes in LEP and ADIPOQ networks. Expression values of 27 shared genes regulated in LEP and ADIPOQ signaling networks are shown in patients treated with alpha1-oleate compared to placebo. Blue = downregulated, red = upregulation.

## Slide 10
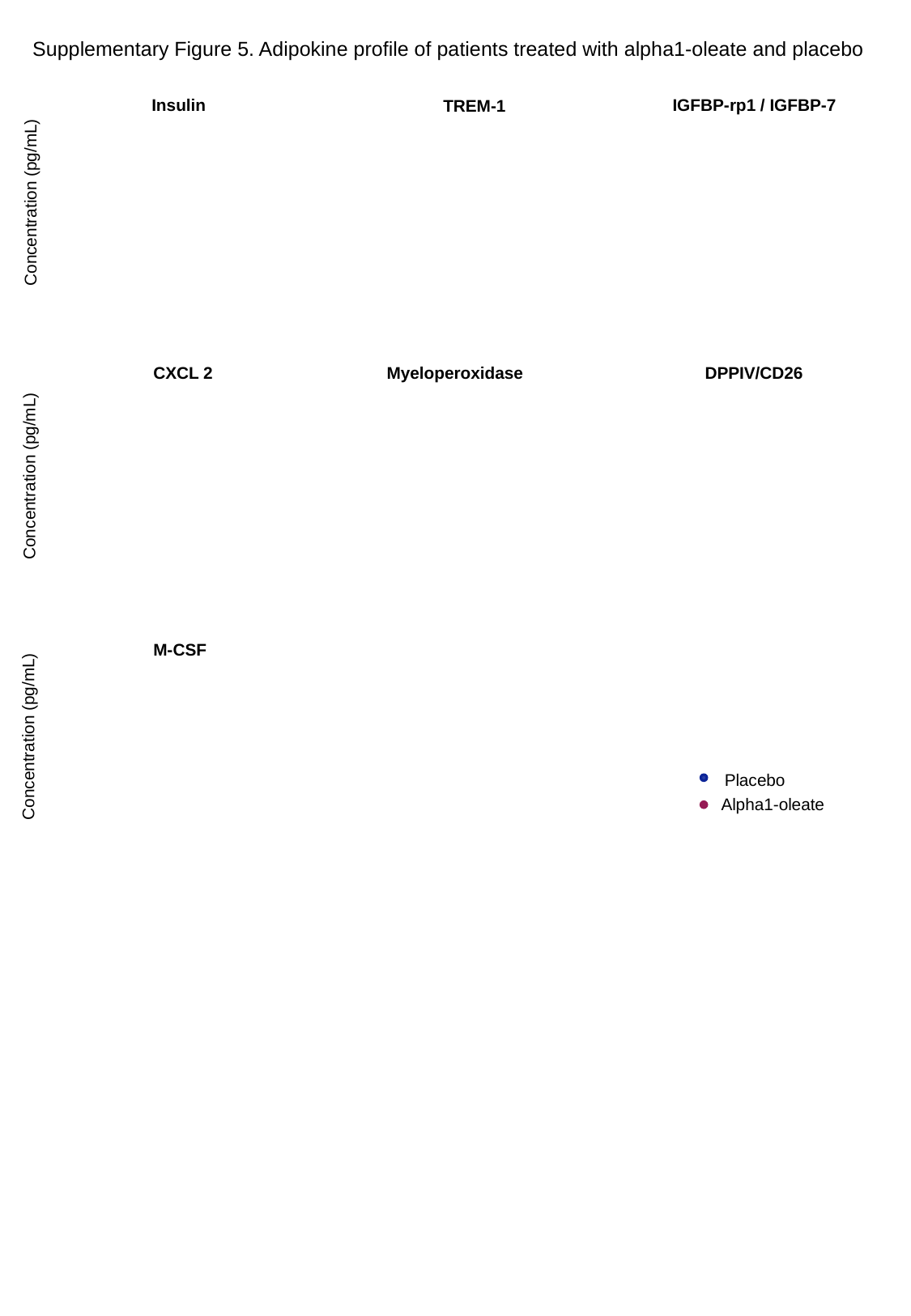

Supplementary Figure 5. Adipokine profile of patients treated with alpha1-oleate and placebo
IGFBP-rp1 / IGFBP-7
Insulin
TREM-1
Concentration (pg/mL)
CXCL 2
Myeloperoxidase
DPPIV/CD26
Concentration (pg/mL)
M-CSF
Concentration (pg/mL)
Placebo
Alpha1-oleate

## Slide 11
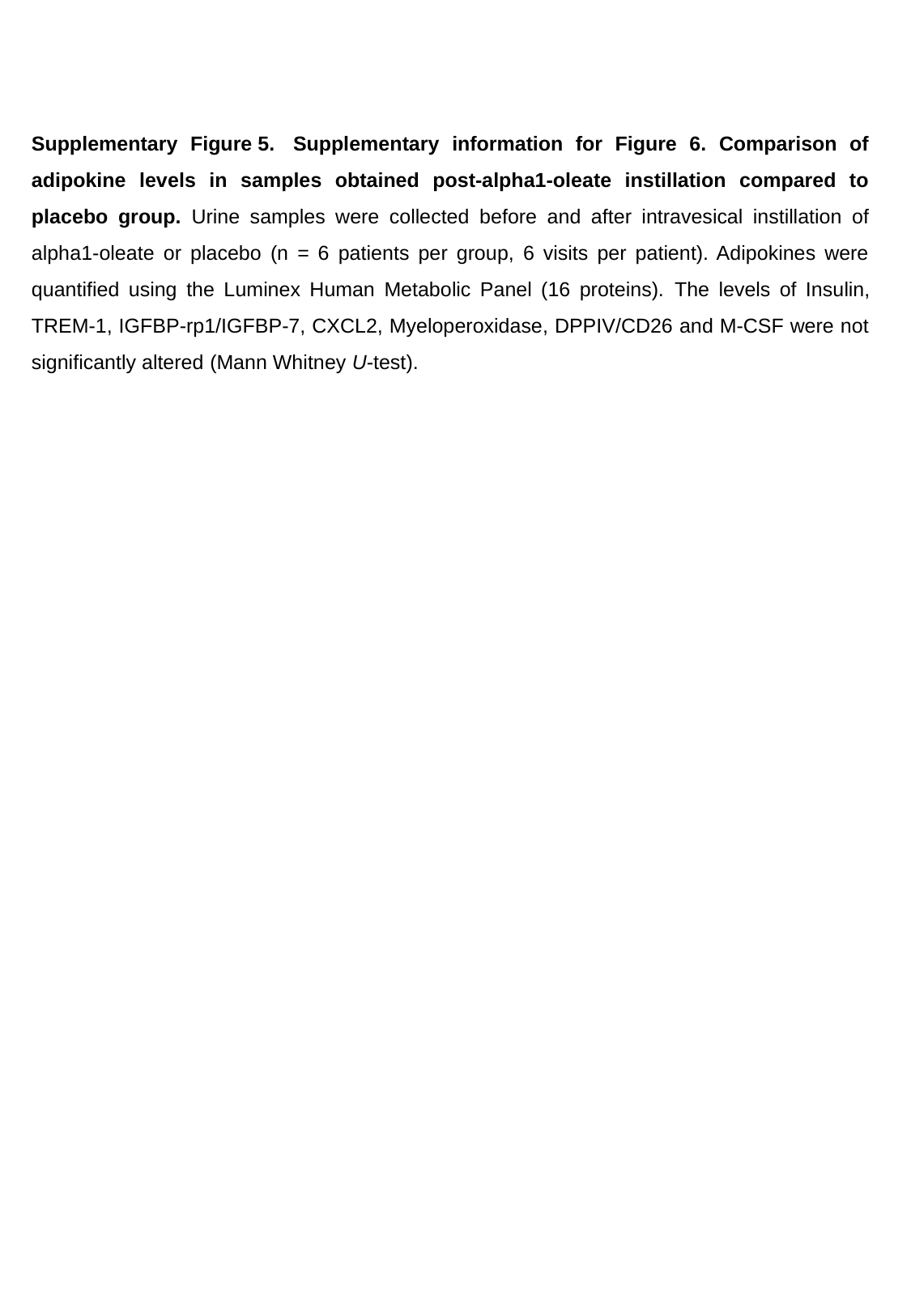

Supplementary Figure 5.  Supplementary information for Figure 6. Comparison of adipokine levels in samples obtained post-alpha1-oleate instillation compared to placebo group. Urine samples were collected before and after intravesical instillation of alpha1-oleate or placebo (n = 6 patients per group, 6 visits per patient). Adipokines were quantified using the Luminex Human Metabolic Panel (16 proteins). The levels of Insulin, TREM-1, IGFBP-rp1/IGFBP-7, CXCL2, Myeloperoxidase, DPPIV/CD26 and M-CSF were not significantly altered (Mann Whitney U-test).

## Slide 12
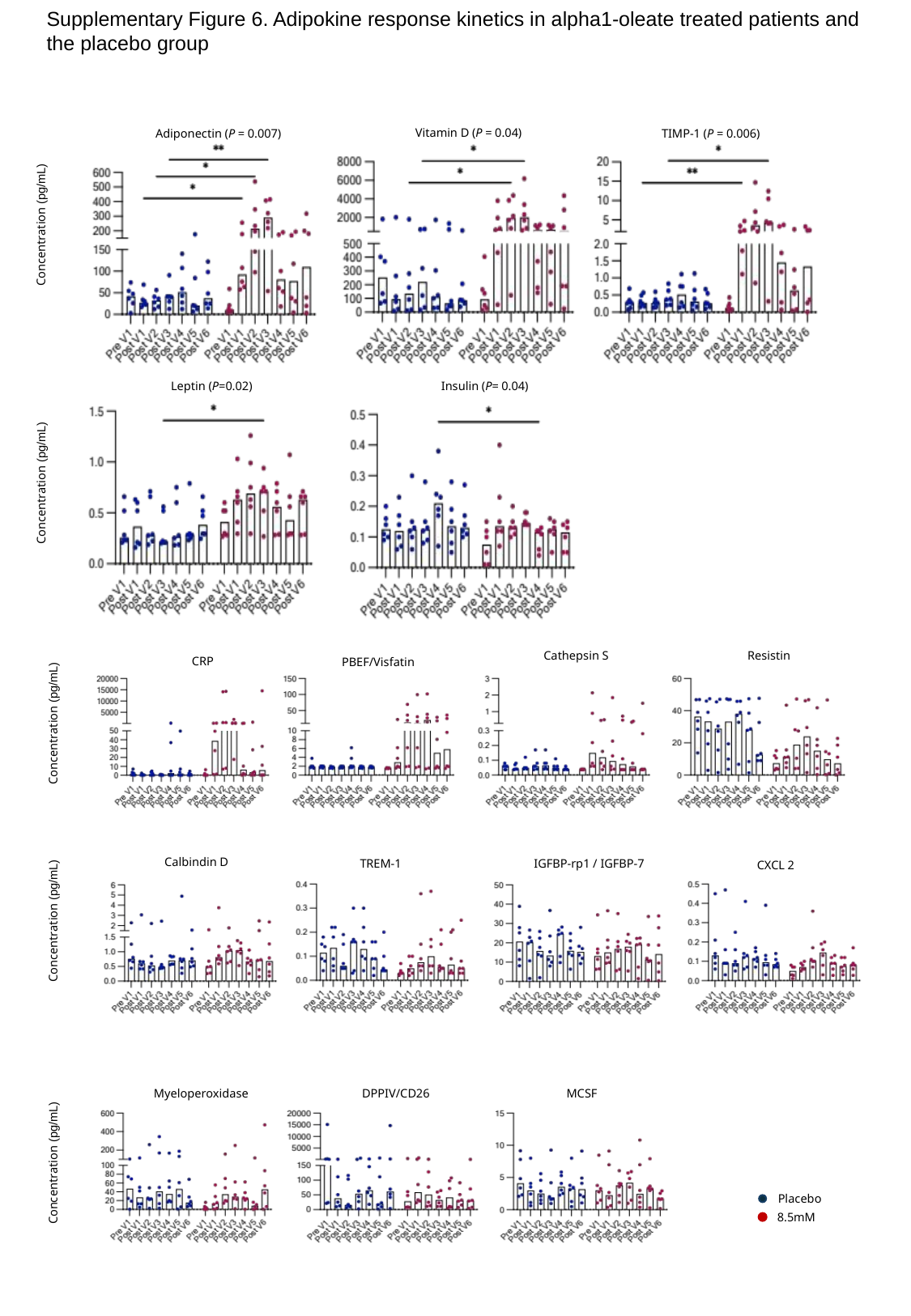

Supplementary Figure 6. Adipokine response kinetics in alpha1-oleate treated patients and the placebo group
Vitamin D (P = 0.04)
TIMP-1 (P = 0.006)
Adiponectin (P = 0.007)
Concentration (pg/mL)
Leptin (P=0.02)
Insulin (P= 0.04)
Concentration (pg/mL)
Cathepsin S
Resistin
CRP
PBEF/Visfatin
Concentration (pg/mL)
Calbindin D
IGFBP-rp1 / IGFBP-7
TREM-1
CXCL 2
Concentration (pg/mL)
DPPIV/CD26
MCSF
Myeloperoxidase
Concentration (pg/mL)
Placebo
 8.5mM

## Slide 13
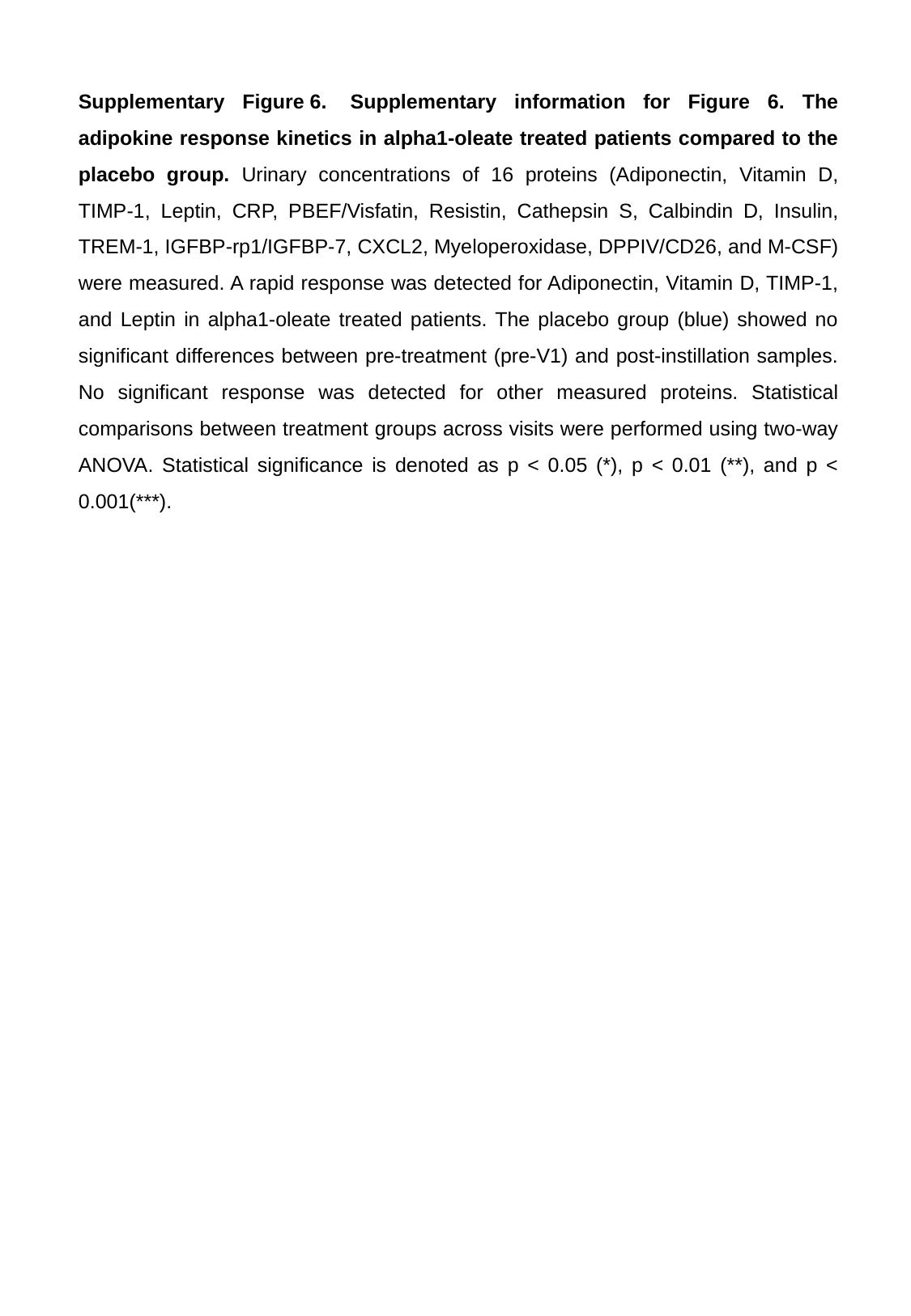

Supplementary Figure 6.  Supplementary information for Figure 6. The adipokine response kinetics in alpha1-oleate treated patients compared to the placebo group. Urinary concentrations of 16 proteins (Adiponectin, Vitamin D, TIMP-1, Leptin, CRP, PBEF/Visfatin, Resistin, Cathepsin S, Calbindin D, Insulin, TREM-1, IGFBP-rp1/IGFBP-7, CXCL2, Myeloperoxidase, DPPIV/CD26, and M-CSF) were measured. A rapid response was detected for Adiponectin, Vitamin D, TIMP-1, and Leptin in alpha1-oleate treated patients. The placebo group (blue) showed no significant differences between pre-treatment (pre-V1) and post-instillation samples. No significant response was detected for other measured proteins. Statistical comparisons between treatment groups across visits were performed using two-way ANOVA. Statistical significance is denoted as p < 0.05 (*), p < 0.01 (**), and p < 0.001(***).

## Slide 14
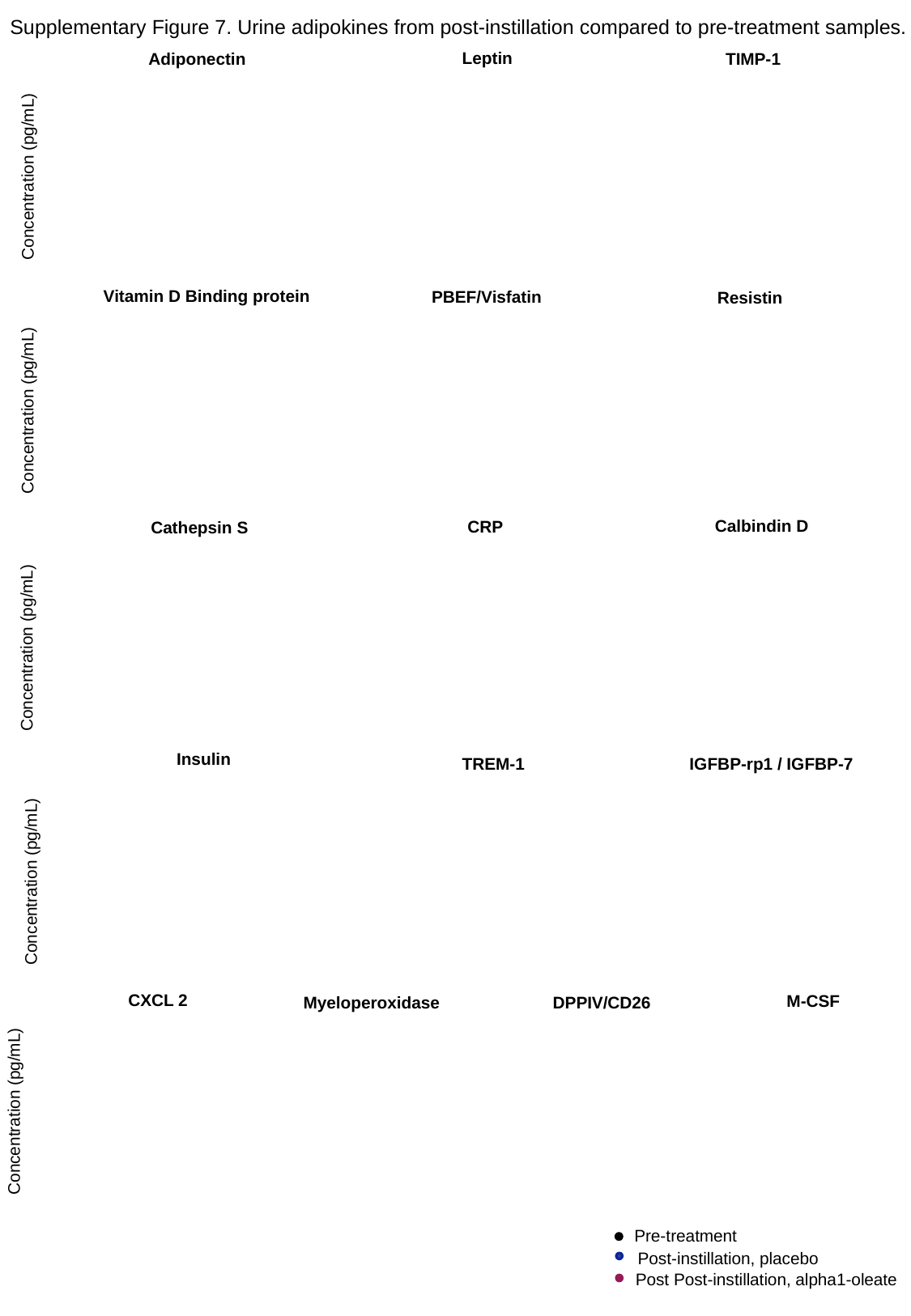

Supplementary Figure 7. Urine adipokines from post-instillation compared to pre-treatment samples.
Leptin
Adiponectin
TIMP-1
Concentration (pg/mL)
Vitamin D Binding protein
PBEF/Visfatin
Resistin
Concentration (pg/mL)
Calbindin D
CRP
Cathepsin S
Concentration (pg/mL)
Insulin
IGFBP-rp1 / IGFBP-7
TREM-1
Concentration (pg/mL)
CXCL 2
M-CSF
Myeloperoxidase
DPPIV/CD26
Concentration (pg/mL)
 Pre-treatment
Post-instillation, placebo
 Post Post-instillation, alpha1-oleate

## Slide 15
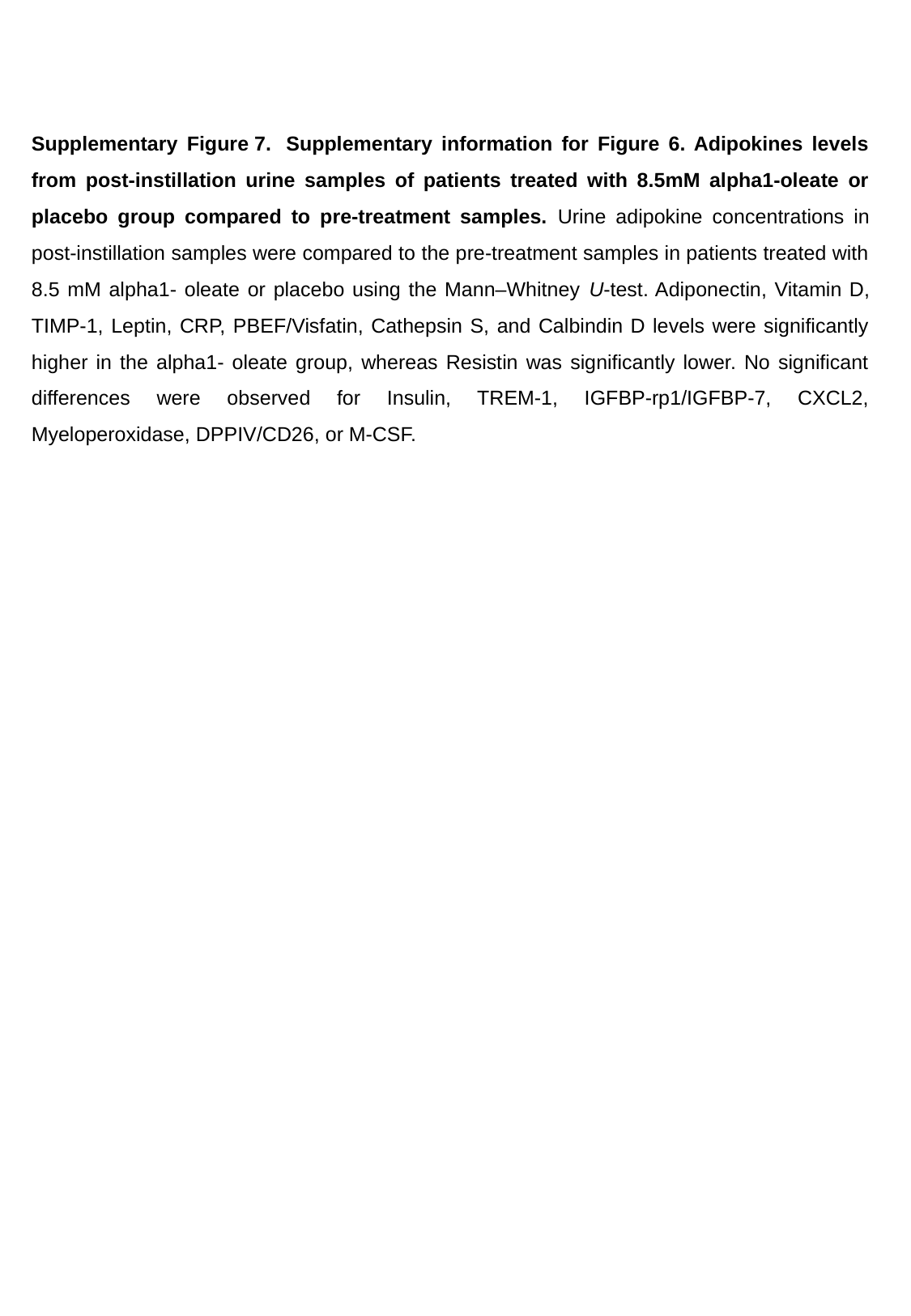

Supplementary Figure 7.  Supplementary information for Figure 6. Adipokines levels from post-instillation urine samples of patients treated with 8.5mM alpha1-oleate or placebo group compared to pre-treatment samples. Urine adipokine concentrations in post-instillation samples were compared to the pre-treatment samples in patients treated with 8.5 mM alpha1- oleate or placebo using the Mann–Whitney U-test. Adiponectin, Vitamin D, TIMP-1, Leptin, CRP, PBEF/Visfatin, Cathepsin S, and Calbindin D levels were significantly higher in the alpha1- oleate group, whereas Resistin was significantly lower. No significant differences were observed for Insulin, TREM-1, IGFBP-rp1/IGFBP-7, CXCL2, Myeloperoxidase, DPPIV/CD26, or M-CSF.

## Slide 16
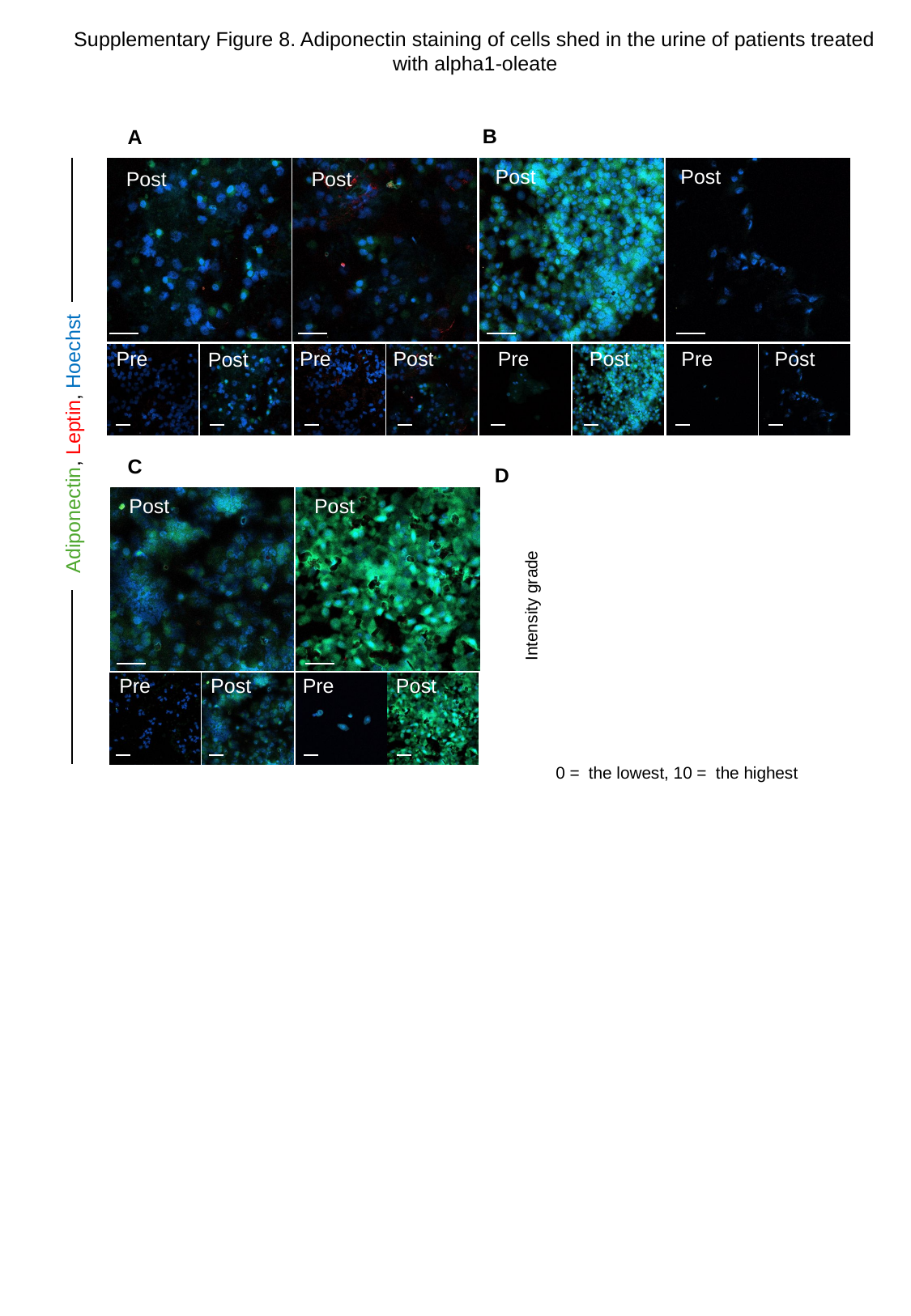

Supplementary Figure 8. Adiponectin staining of cells shed in the urine of patients treated with alpha1-oleate
B
A
Post
Post
Post
Post
Pre
Pre
Post
Pre
Post
Pre
Post
Post
Adiponectin, Leptin, Hoechst
C
D
Post
Post
Pre
Pre
Post
Post
Intensity grade
0 = the lowest, 10 = the highest

## Slide 17
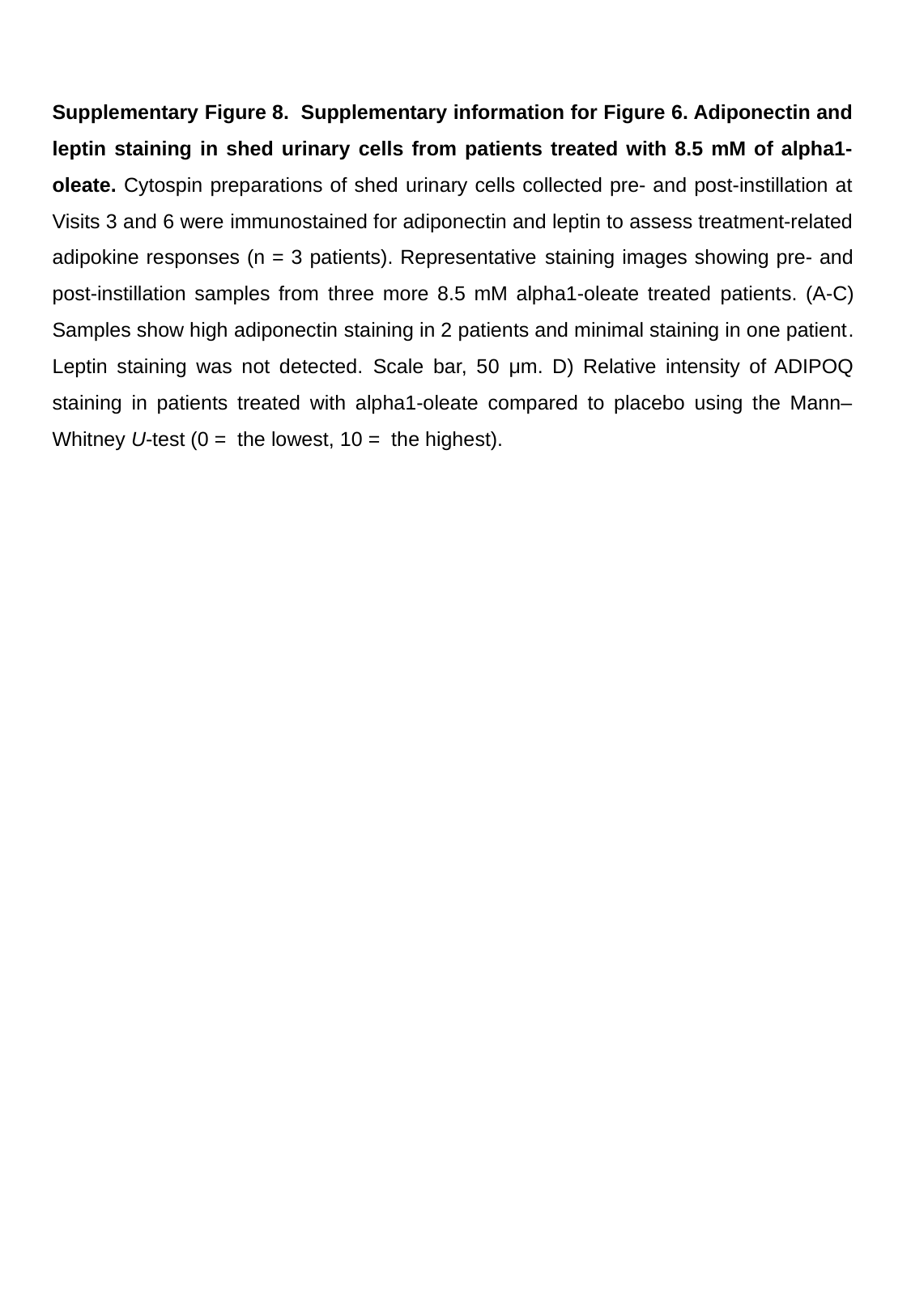

Supplementary Figure 8.  Supplementary information for Figure 6. Adiponectin and leptin staining in shed urinary cells from patients treated with 8.5 mM of alpha1- oleate. Cytospin preparations of shed urinary cells collected pre- and post-instillation at Visits 3 and 6 were immunostained for adiponectin and leptin to assess treatment-related adipokine responses (n = 3 patients). Representative staining images showing pre- and post-instillation samples from three more 8.5 mM alpha1-oleate treated patients. (A-C) Samples show high adiponectin staining in 2 patients and minimal staining in one patient. Leptin staining was not detected. Scale bar, 50 μm. D) Relative intensity of ADIPOQ staining in patients treated with alpha1-oleate compared to placebo using the Mann–Whitney U-test (0 = the lowest, 10 = the highest).

## Slide 18
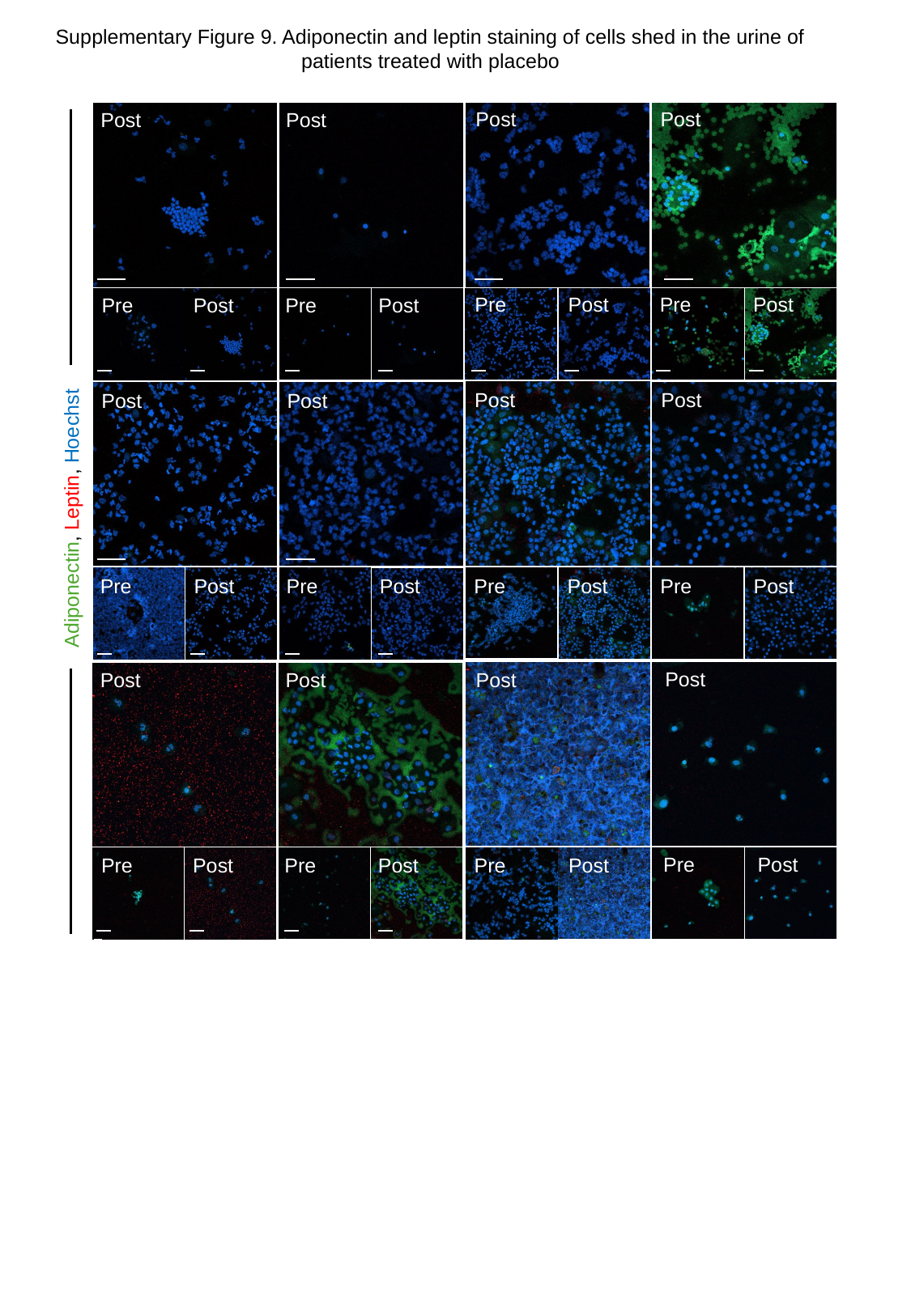

Supplementary Figure 9. Adiponectin and leptin staining of cells shed in the urine of patients treated with placebo
Post
Post
Post
Post
Post
Pre
Post
Pre
Post
Pre
Pre
Post
Post
Post
Post
Post
Post
Pre
Post
Pre
Post
Pre
Post
Pre
Post
Post
Post
Post
Post
Pre
Post
Pre
Post
Pre
Post
Pre
Post
Post
Post
Post
Pre
Post
Pre
Post
Pre
Pre
Post
Post
Post
Post
Post
Post
Pre
Adiponectin, Leptin, Hoechst
Post
Pre
Pre
Post
Pre
Post
Pre
Post
Pre
Post
Pre
Post
Post
Post
Post
Post
Pre
Post
Pre
Pre
Post
Pre
Post
Post
Pre

## Slide 19
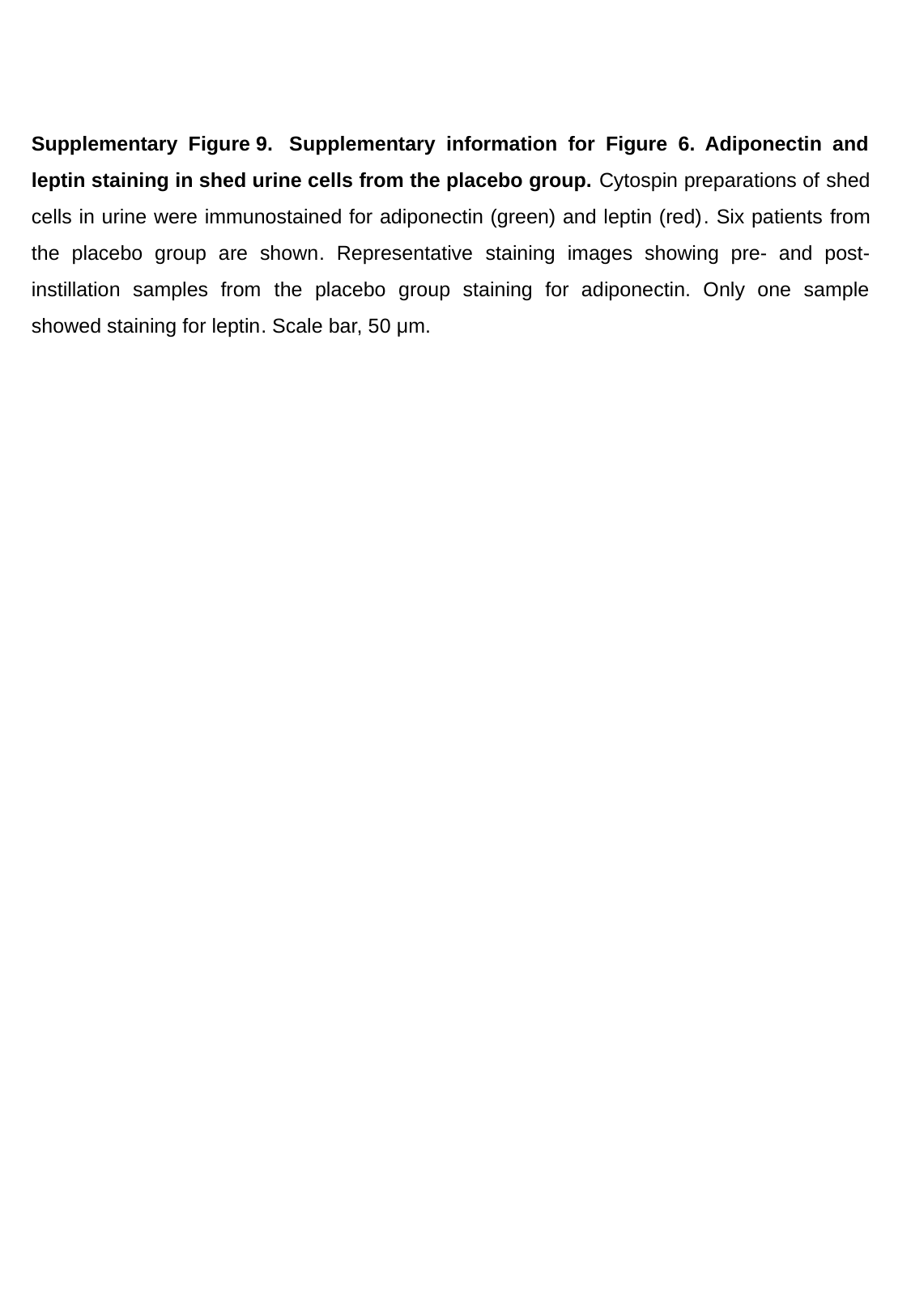

Supplementary Figure 9.  Supplementary information for Figure 6. Adiponectin and leptin staining in shed urine cells from the placebo group. Cytospin preparations of shed cells in urine were immunostained for adiponectin (green) and leptin (red). Six patients from the placebo group are shown. Representative staining images showing pre- and post-instillation samples from the placebo group staining for adiponectin. Only one sample showed staining for leptin. Scale bar, 50 μm.
